# Immersive Interactive Technologies for Positive Change: A Scoping Review and Design Considerations

**DOI:** 10.3389/fpsyg.2018.01354

**Published:** 2018-08-03

**Authors:** Alexandra Kitson, Mirjana Prpa, Bernhard E. Riecke

**Affiliations:** iSpaceLab, School of Interactive Arts and Technology, Simon Fraser University, Surrey, BC, Canada

**Keywords:** scoping review, immersive technology, positive technology, transformative technology, design, virtual reality, augmented reality, mixed reality

## Abstract

Practices such as mindfulness, introspection, and self-reflection are known to have positive short and long-term effects on health and well-being. However, in today's modern, fast-paced, technological world tempted by distractions these practices are often hard to access and relate to a broader audience. Consequently, technologies have emerged that mediate personal experiences, which is reflected in the high number of available applications designed to elicit positive changes. These technologies elicit positive changes by bringing users' attention to the self—from technologies that show representation of quantified personal data, to technologies that provide experiences that guide the user closer in understanding the self. However, while many designs available today are either built to support or are informed by these aforementioned practices, the question remains: how can we most effectively employ different design elements and interaction strategies to support positive change? Moreover, what types of input and output modalities contribute to eliciting positive states? To address these questions, we present here a state of the art scoping review of immersive interactive technologies that serve in a role of a mediator for positive change in users. We performed a literature search using ACM Digital Library, Web of Science, IEEE Xplore, and Design and Applied Arts Index (beginning of literature—January 1, 2018). We retrieved English-language articles for review, and we searched for published and unpublished studies. Risk of bias was assessed with Downs and Black 26-item QAT scale. We included 34 articles as relevant to the literature, and the analysis of the articles resulted in 38 instances of 33 immersive, interactive experiences relating to positive human functioning. Our contribution is three-fold: First we provide a scoping review of immersive interactive technologies for positive change; Second, we propose both a framework for future designs of positive interactive technologies and design consideration informed by the comparative analysis of the designs; Third, we provide design considerations for immersive, interactive technologies to elicit positive states and support positive change.

## Introduction

**Figure 1:**
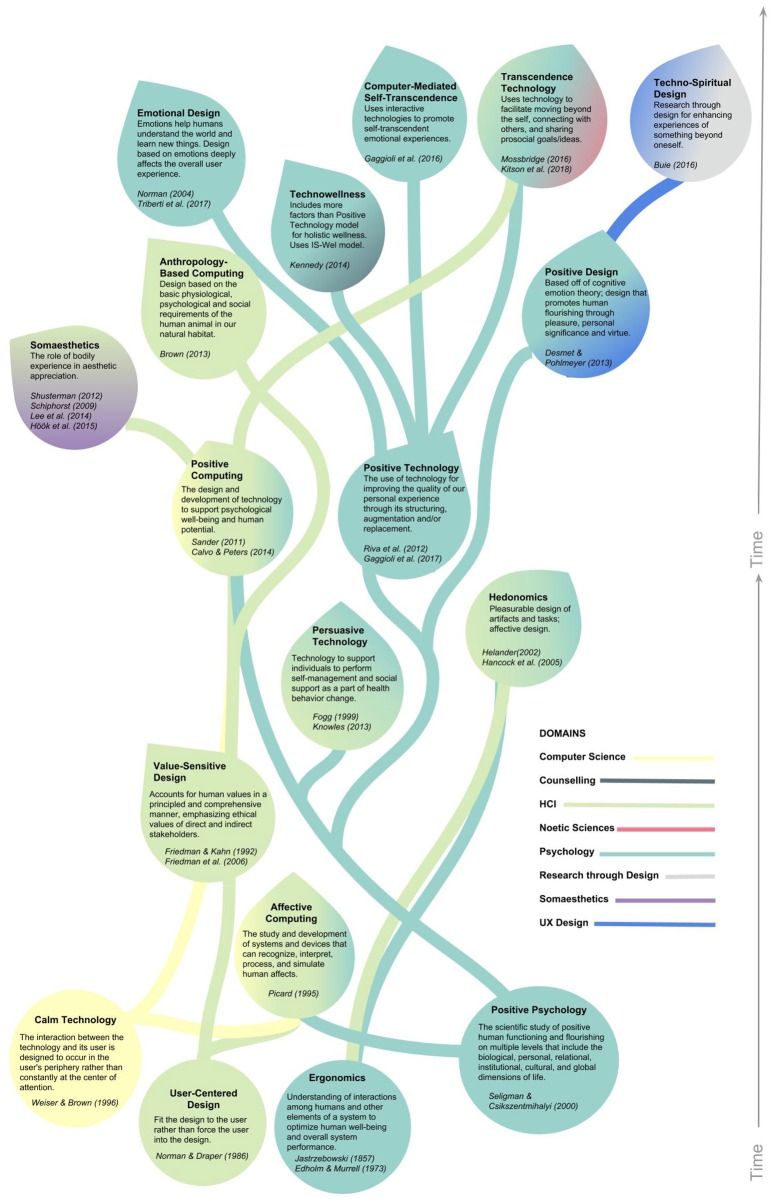
Existing domains of technology for positive functioning and well-being: moving along the y-axis is the passage of time on a non-linear scale that depicts the growth of different fields that stem from the foundational three domains of Computer-Science, HCI, and Psychology. Each color represents a different domain; the stems show the progression of the domain, feeding into the next; and the leaves are colored by the influences from those domains. Leaves represent the first conceptualization of an approach, and do not imply the cessation of progress, e.g., Affective Computing was first introduced in the 1990s and is still relevant today.

Technology is becoming increasingly more prevalent in our everyday lives. Yet, for all the new hardware and gadgets available, we have only recently seen an increased interest in designers, developers, and researchers deliberately thinking about how these technologies might be used to improve our lives and increase our well-being (Bowman and McMahan, 2007; Roo et al., 2016; Valmaggia et al., 2016; Gaggioli et al., 2017). The Western practice and literature so far has focused primarily on mental health problems and treatments, from a medical or psychiatric lens (Valmaggia et al., 2016) and with a focus on treating symptoms rather prevention. Furthermore, literature focusing on healthy populations and using a preventative medicine point of view is uncommon. Focusing on preventable measures is important because non-communicable diseases cause 70% of deaths globally and about half of all deaths in the US were preventable (Mokdad et al., 2004; WHO, The top 10 causes of death), and the use of preventative healthcare has shown to provide numerous health benefits and increase quality of life dramatically (Cohen et al., 2008; Maciosek et al., 2010). That said, there does appear to be a rise in interest in using technology for positive human functioning and well-being across many different domains. This diversified interest seems to imply promise for future applications of technology for improving positive experiences and health. Yet, a challenge lies in trying to integrate all the existing knowledge across the various domains because, although they are all aiming toward a common goal, they are using different terminology, frameworks, and theoretical lenses to approach the topic. We have created a visualization in an attempt to better understand both the development of these different domains over time and how they interact with each other (see Figure 1), and will elaborate on it below. Approaches to technology that supports positive human functioning and well-being appear to be seeded from three different domains: Psychology, HCI, and Computer Science. We will briefly discuss the history of these approaches, although we recognize that this may not be an exhaustive list because of the highly multidisciplinary nature of this research area.

### Psychology

In the late 1990s, Psychology was dominated by psycho-analysis and behaviorism that focused on a “mental illness” model of human functioning. Positive Psychology was then introduced as a counter to this way of thinking; instead it emphasized happiness, well-being, and positivity. Positive Psychology originated with Seligman's PERMA theory and Csikszentmihalyi's Flow theory. PERMA consists of five elements that can help people reach a life of fulfillment, happiness, and meaning: Positive emotions, Engagement, Relationships, Meaning, and Achievement. Flow is an activity with goals/progress, feedback, and balancing perceived challenge and skill. Together, these two theories then formed the basis of several positive technology approaches including Persuasive Technology, Positive Computing, and Positive Technology. Fogg (1999) was one of the first researchers to put forth the idea that computers are able to persuade or change the behavior of people. Later, Knowles (2013) expanded upon this idea by placing importance on implicit values of both the user and designer to motivate behavior change. Positive Computing and Positive Technology both arose as ideas around the same time and are highly related (Gaggioli et al., 2017). Both consider ways for bringing well-being considerations into interaction design through positive technology theories. One difference is that Positive Computing (Sander, 2011; Calvo and Peters, 2014) uses an engineering lens for considering well-being in any technology either as preventative or active integration, whereas Positive Technology (Riva et al., 2012) uses a psychological lens for considering technology as a platform for supporting and sustaining well-being and the process of change. TechnoWellness (Kennedy, 2014) emerged in response to Positive Technology, arguing that Positive Technology was missing key factors for holistic wellness based on a counseling perspective that uses the IS-Wel model (Myers and Sweeney, 2005). The IS-Wel model, or Indivisible Self Model of Wellness, integrates both the model of the Indivisible-Self and the five factor Wellness Wheel. Emotional Design emerged with this effort to promote positive emotions or pleasure in users (Norman, 2004), and has since been expanded upon to the design of interactive technologies (Triberti et al., 2017). Directly stemming from Positive Technology came Computer-Mediated Self-Transcendence (Gaggioli et al., 2016), which is a more specific pathway of Positive Technology that focuses on interactive technologies to support self-transcendent emotional experiences. Similar to Computer-Mediated Self-Transcendence, Transcendence Technology (Mossbridge, 2016) seeks to use technology to move beyond the self and connect with others, but was developed more through a noetic sciences, i.e., parapsychological, lens. A specific example of Transcendence Technology is the study of lucid dreaming to inform the design of virtual reality introspective experience (Kitson et al., 2018). Desmet and Pohlmeyer (2013) took cognitive emotion theory and combined it with user experience (UX) design to form the framework of Positive Design, design that promotes human flourishing. A few years later, Buie (2016) formed Techno-Spiritual Design. Seemingly not wanting to use any of the existing theories on designing technology for well-being, Buie used a research through design approach to actively engage the user throughout the design process of creating technological experiences that support going beyond the self.

### Human-computer interaction (HCI) and computer science

The idea of understanding human nature in relation to work has been around for a long time. In 1857, Jastrzebowski (1857) first coined the term ergonomics, referring to worker productivity in labor, entertainment, reasoning, and dedication. More contemporarily, ergonomics was reintroduced in the 1970s by Murrell (Edholm and Murrell, 1973) to mean understanding human-system interactions to optimize human well-being and system performance. Ergonomics then took on many different forms and specialties including cognitive ergonomics that encompasses usability, human-computer interaction (HCI), and user experience (UX) design. Some researchers viewed Ergonomics as focusing on negatively framed constructs such as pain prevention, particularly in the workplace. In order to look at the same human-technology interaction problem from a different perspective, a group of researchers created Hedonomics, the science and design devoted to the promotion of pleasurable human-technology interaction (Helander, 2002; Hancock et al., 2005). In the mid 1980s, the term User-Centered Design was first coined by Donald A. Norman's work in their lab (Norman and Draper, 1986). This concept focused on putting the user's needs and wants at the forefront of the product rather than trying to force the user to adapt their existing behaviors. User-Centered Design was soon adopted into many fields as a way to incorporate user feedback throughout the design process and not only at the evaluation phase as was originally used. Friedman and Kahn (1992) introduced Value-Sensitive Design—developing technology by making decisions based on implicit and explicit values, and that values of both designers and users should be accounted for. Value-Sensitive Design guidelines were eventually developed with an ethical values framework in mind (Friedman et al., 2006). Meanwhile, in the domain of computer science, Weiser and Brown (1996) were developing a framework for designing the interaction between technology and user that had the technology seamlessly integrated without constantly being at the center of attention—Calm Technology. At the same time, Affective Computing used both physiological and psychological theories and both computer science and HCI lenses to support the design of technology that recognizes, interprets, processes, and simulate human affect (Picard, 2010). The seeds of both Computer Science and HCI contributed to fields of Positive Computing and Persuasive Technology as well (discussed above). Two other fields that emerged from the domain of HCI are Somaesthetics (Schiphorst, 2009; Shusterman, 2012) and Anthropology-Based Computing (Brown, 2013). Somaesthetics grounds itself in human bodily experience and movement to inform design, particularly the aesthetics of interaction. This approach has been adopted by many designers of technologies that support positive human functioning (for example: Lee et al., 2014; Höök et al., 2015). Anthropology-Based Computing uses basic human behavior in our natural habitat as a basis of designing technological systems.

### Motivation

Overall, following the emergence and the advances in the field of human-computer interaction, many different research domains have been focusing on designing for human-technology interactions that support positive human functioning and well-being, as discussed above. The foci of HCI research have been greatly concerned with the question: How to aid and mediate the interaction between a user and a system in such a way to allow for more efficient accomplishment of a task, that being retrieving the information, or alleviating states (e.g., stress) that can prevent them from accomplishing a task. Furthermore, early technological developments were focused on performance and production from an Engineering and Computer Science standpoint of usability and information retrieval. With the advent of the informational age, HCI and Psychological theories came together to ground human-technology interactions in genuine human experience, emphasizing the stance of the user over the system. We can see these ideas and framework permeate into the UX and design space, leading to current trends of using immersive, interactive technologies for providing experiential accounts mediated through technologies that support positive human functioning and well-being. However, there is not a clear understanding of what this design space looks like and how we might move forward with all these approaches in mind. In continuing the trajectory laid out in Figure 1, we seek to understand how immersive, interactive technologies might elicit positive states and support positive change. We found that there exist a few review articles on interactive technologies for supporting mindfulness (Sliwinski et al., 2017), transcendence (Mossbridge, 2016), and health (Botella et al., 2017). However, there does not seem to be comprehensive reviews looking at immersive, interactive technologies in eliciting positive states and supporting positive change. This motivated us to perform a scoping review in order to explore the extent of the literature in this domain, and potentially inform the scope of a future systematic review. While mindfulness may fit into the idea of positive states and positive change, we differentiate ourselves by expanding and including *immersive, interactive experiences that support people on hedonic, eudaimonic, and social/interpersonal levels*, which are the three positive technology domains as put forth by Riva et al. (2012). Hedonic relates to pleasant sensations, eudaimonic focuses on meaning and self-realization, and social/interpersonal involves relations or communications between people. We emphasize the focus on immersive technologies because they have a high potential of influencing and transforming the user through increased presence, the physical feeling of being in the simulated environment, which then enhances the experience's effectiveness (Riva et al., 2007; Diemer et al., 2015; Cummings and Bailenson, 2016).

### Defining immersive, interactive, and well-being

The term “immersion” has been discussed and used by researchers in the technology field for decades, yet there seems to still be some confusion because the term is so widely used to describe experiences in games (Brown and Cairn's, 2004; Ermi and Mäyrä, 2005), paintings (Grau, 2003), literature (Nell, 1988), and cinema (Bazin, 1967). Defining immersion is critical to our understanding of the relationship between the user and the virtual environment because it addresses the very notion of being in the context of such simulated environments (Grimshaw, 2013). Moreover, without a clear definition of the concept, results can be uninterpretable. Some researchers, particularly in the gaming field, view immersion as different facets: sensory-motoric immersion, cognitive immersion, emotional immersion, and spatial immersion (Bjork and Holopainen, 2005). Ermi and Mäyrä's (2005) SCI model of immersion consists of three components: sensory, challenge-based, and imaginative. These models of immersion seem to suggest that immersion is a psychological process. However, contemporary researchers of immersion (IJsselsteijn and Riva, 2003; Rettie, 2004; van den Hoogen et al., 2009) roughly follow Slater and Wilbur's definition of system immersion as

a description of a technology… that describes the extent to which the computer displays are capable of delivering an inclusive, extensive, surrounding and vivid illusion of reality to the sense of a human participant (Slater and Wilbur, 1997, p. 606).

Here, immersion appears to be less of a psychological process and more of a physical process where our bodies and senses are tricked into behaving and reacting like the virtual environment is real. A similar construct, presence, is then the psychological process of believing the virtual environment is real or the feeling of “being there” (IJsselsteijn and Riva, 2003). Following these definitions of immersion and presence, virtual lucidity, a term defined by Quaglia and Holecek (2018) is when a person is immersed (the virtual environment feels real) yet not present (knows the virtual environment is not real). This review is focused on the psychological factors determining presence; we note, however, that there are different theoretical accounts on the definition of presence and which factors influence it (Coelho et al., 2006; Triberti and Riva, 2016). Aligning ourselves with contemporary definitions, we also chose to follow Slater and Wilbur's definition of immersive as an objective property of the technology for the purposes of this review.

Steuer (1992) defines interactivity as “the extent to which users can participate in modifying the form and content of a mediated environment in real time” (p. 14). Rubio-Tamayo, Barrio, and García have defined interactivity as

the potential to receive information from the ensemble of our senses and to construct and configure an alternate reality or to simulate reality. Additionally, it is the potential to influence (in real time) in the digital environments, the objects and the narrative framed in it (Rubio-Tamayo et al., 2017, p. 11).

Non-interactive technological experiences such as web-pages, video instructions, guided mobile apps, 360 videos, and movies were excluded from this review. These applications can provide, from a certain point of view, a two-way flow of information between computer and user. However, they do not meet the definition proposed by Rubio-Tamayo et al. (2017) as having the potential to influence digital environments. Related to interactivity is the construct of embodiment, where cognition is shaped by the body (Varela et al., 1992; Markman and Brendl, 2005). Technologies can be embodied for their abilities to modify the cognitive factors regulating the experience of body and space (Riva et al., 2016).

Well-being refers generally to the interconnected dimensions of physical, mental, and social health of an individual. A few models in psychology attempt to understand and define well-being. First, is the *broaden-and-build hypothesis* that states positive emotions broaden people's momentary thought-action repertoires, and positive emotions build over time enduring psychological, intellectual, physical, and social resources (Fredrickson, 2001). Second, is the *self-determination theory* where autonomy, competence, and relatedness needs must be satisfied in order to foster well-being and health; and self-determined behavior is intrinsically motivated (Ryan and Deci, 2000). Third, *authentic happiness theory* postulates that pleasant life, engaged life, and meaningful life are the three concepts needed for well-being (Seligman, 2002). However, several limitations were found with this theory, and so he developed *PERMA-theory* (Seligman and Csikszentmihalyi, 2014): **P**ositive emotions (happiness, joy, excitement, satisfaction, pride, awe); **E**ngagement (flow); **R**elationships (work, familial, romantic, platonic); **M**eaning (purpose); **A**ccomplishments (success and mastery). In this review, we consider all of these conceptualizations of well-being in an attempt to discover as many immersive, interactive experiences that support well-being as possible.

### Objectives and research questions

#### We make four contributions in this paper

First, we identify the design elements and interaction strategies that contribute to well-being and positive affective states. And, in this process, we unveil design nuances and note the obstacles users encounter in interacting with the particular XR technology, a term which includes virtual, augmented, and mixed realities. Second, we identify the input-output modalities of the system and the psychological outcomes of each study. Third, we present a framework for designing transformative experiences with immersive, interactive technologies whose goal is to elicit positive states and support positive human change. Fourth, we provide design considerations informed by the comparative analysis of the designs and a framework for future designs of positive interactive technologies with the goal of eliciting positive states and supporting positive change in users.

To assess the current state of the research in positive, immersive, interactive technologies, this scoping literature review will address two research questions:

RQ1: How can we most effectively employ different design elements and interaction strategies to support positive change in immersive, interactive technologies?
RQ2: What are the input and output modalities of immersive, interactive technologies that contribute to eliciting positive states?

## Methods

### Scoping review protocol

We undertook this study as a scoping literature review based on guidelines proposed by Arksey and O'Malley (2005) and Levac et al. (2010). Scoping reviews are a process of mapping the existing literature or evidence base in order to identify research gaps and summarize research findings. Scoping reviews differ from systematic reviews in that they use broader research questions, inclusion/exclusion criteria can be developed post hoc, quality is not an initial priority, it may or may not involve data extraction, and synthesis is more qualitative and not typically quantitative (Armstrong et al., 2011). Still, both scoping and systematic reviews require rigor and time to complete. We decided on a scoping review over a systematic review because our research questions are explorative and our objective is to map the literature with a broad viewpoint, rather than to respond to narrow research questions. We registered our review on PROSPERO—registration # CRD42018082752. The following steps were taken in accordance to the scoping guidelines:

1. Identify the research questions,
2. Identify relevant studies,
3. Study selection,
4. Charting the data,
5. Collating, summarizing, and reporting results.

### Identifying relevant studies

A systematic search of the literature was performed in four academic databases that were considered the most relevant due to their focus on HCI: ACM Digital Library, Web of Science, Design and Applied Arts Index (DAAI), and IEEE Xplore (IEEE/IET Electronic Library). Google Scholar was used as an additional academic search engine to ensure all relevant articles were found.

The search was focused on immersive and interactive technologies and experiences, which included virtual, augmented, and mixed realities, otherwise known as “XR.” The XR experiences were related to positive well-being and not on clinical interventions relating to treating disease. We sought articles from any time until January 2018, the end of this search. We utilized the retrieval of relevant articles with the following search terms based on the definitions of immersive, interactive, and well-being for technologies:

(“immersive” OR “interactive” OR “virtual realit^*^” OR “augmented realit^*^” OR “mixed realit^*^” OR “extended realit^*^”) **AND** (“well-being” OR “wellbeing” OR “well-being” OR “wellness” OR “positive” OR “emotion^*^” OR “social” OR “autonomy” OR “competence” OR “relatedness” OR “pleasant” OR “engag^*^” OR “meaning^*^” OR “happiness” OR “joy” OR “excitement” OR “satisfaction” OR “pride” OR “awe” OR “flow” OR “relationship^*^” OR “purpose” OR “success” OR “mastery”).

The first part of the search index includes technologies that are immersive and interactive. The second part includes terms taken directly from well-being theories: broaden-and-build model (Fredrickson, 2001), self-determination theory (Ryan and Deci, 2000), authentic happiness theory (Seligman, 2002), and PERMA theory (Seligman, 2012). We also decided to include the following search terms, which were part of a sub-search, based on the theoretical approaches we described in the introduction and list in Figure 1 because they are directly related to supporting positive human functioning and well-being with technology:

(“tech^*^” OR “computing”) **AND** (“change” OR “support tool” OR “connection” OR “calm^*^” OR “essential self” OR “transcenden^*^” OR “transformative” OR “self-transcend^*^” OR “consciousness hacking” OR “UX for good” OR “spiritual” OR “persuasive” OR “lovingkindness” OR “metta” OR “mindful^*^” OR “meditat^*^”).

We applied this search string to the title, abstract, full-text, and author keywords. Applicable articles were also identified through backward reference searching, i.e., by screening the reference lists of retrieved publications. Google Scholar was utilized for the backward reference searching to run general searches of specific references and to identify relevant articles.

### Study selection

Peer-reviewed articles as well as scholarly work such as dissertations, theses, and conference proceedings with the following characteristics, published from the beginning of the literature until January 2018, were included:

- written in English,
- having at least one immersive and interactive technology,
- having a goal to improve well-being.

We included other scholarly work, i.e., dissertations, theses, and conference proceedings, because these works were also relevant to the field, they often report studies that can be important for our research questions, and we wanted to be comprehensive in our study selection. Blog entries and websites, although can be insightful and managed by scholarly affiliations, were excluded because they often do not report studies and are difficult to compare to other literature types. Immersive, interactive technologies were chosen as the appropriate setups in order to keep the review the most up to date, and because they are relevant for transformative experience design. The immersive, interactive experiences themselves needed to include a well-being component or focus on positive human functioning in order to relate to the core elements of transcendent experiences.

Consequently, articles with the following characteristics were excluded:

- using exclusively desktop-based, tablet-based, or mobile virtual environments,
- non-interactive experiences,
- addressing solely conceptual matters, such as theoretical models, frameworks, reviews, etc.,
- using immersive, interactive technology as a tool for studying a different, unrelated topic.

**Figure 2:**
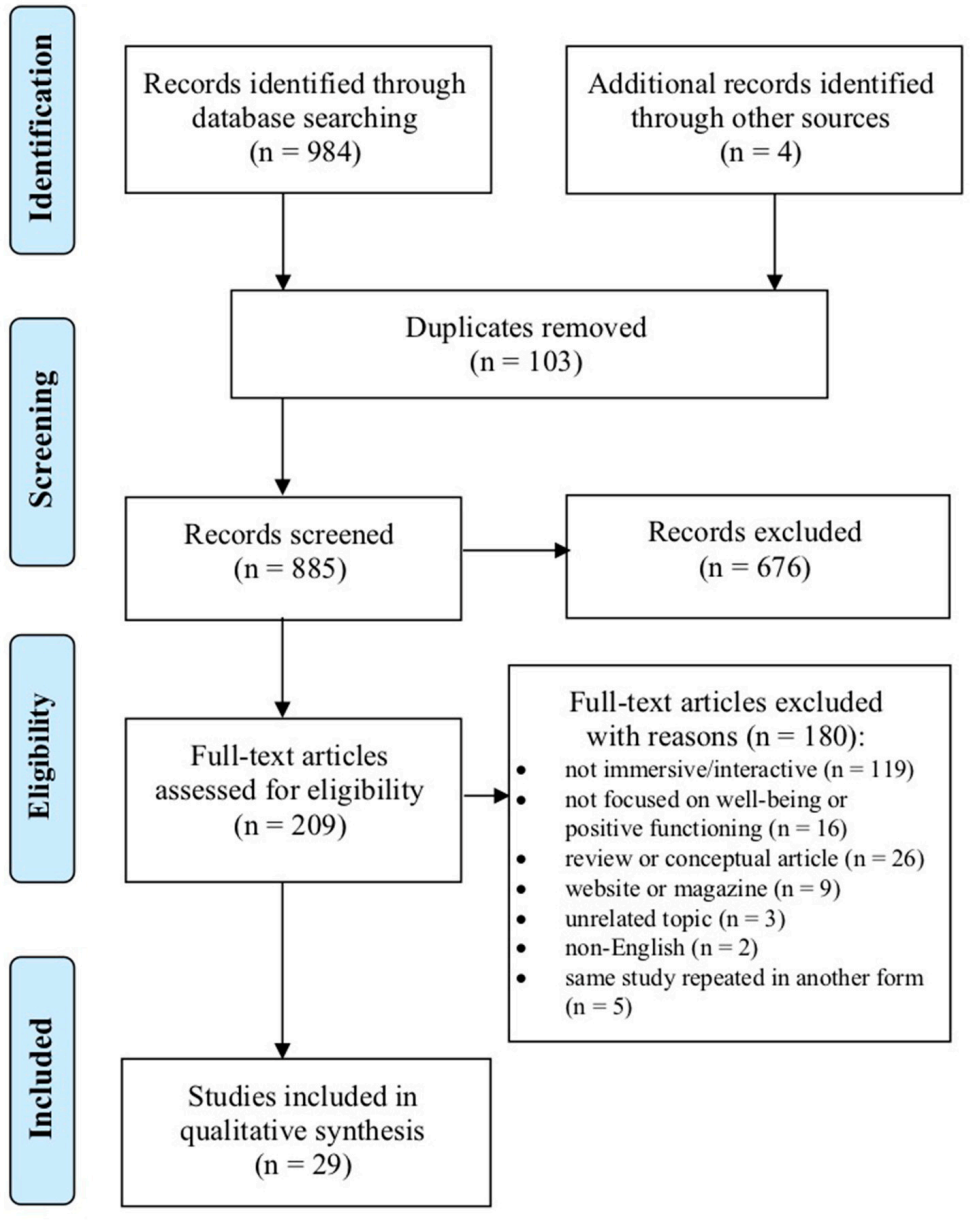
Flowchart of the included and excluded articles from the assessment of screening and eligibility process.

The screening process and its results are visualized in Figure 2. The first and second author screened the results independently and then compared agreement. If there was a discrepancy, then the third author was consulted. The initial search elicited 984 articles from the four databases and four from the reference review, which were retrieved with Google Scholar. One hundred and three duplicates were identified and removed, leaving 885 articles to be screened. The initial screening of studies was based on their abstracts and titles, excluding noticeably irrelevant studies based on the inclusion/exclusion criteria listed prior. In total, 209 articles were identified as appropriate for inclusion, and they were moved to the second screening round. The second round of screening was based on the full text of the articles and the first and second authors independently reviewed each using the inclusion/exclusion criteria set before the search, as suggested by Levac et al. (2010). In total, 29 articles were identified as appropriate for inclusion and relevant to the current review. The authors reviewed all 29 articles independently. All reviewers together conjointly shaped the categories and themes of the review, based on the data extraction process. The authors discussed and settled any disagreements of the qualitative synthesis of the review before writing the final narrative.

### Charting the data

The screening process resulted in 29 articles that satisfied the inclusion criteria. The data extracted from each article were the following:

1. source and full reference
2. description and name of the immersive interactive system
3. relevance to well-being and positive functioning
4. type of XR
5. technology used
6. platform
7. target user
8. number of users in study
9. input/output modalities
10. design elements and interaction strategies used
11. outcome
12. how design elements and interaction strategies contributed to support positive change and/or elicit positive states

See Supplementary Material for a detailed table of the data extracted. If data were missing, the study authors were contacted. The first author performed the data extraction process.

### Collating, summarizing, and reporting the results

The collected data were synthesized by identifying themes emanating from information reported in each accepted paper and related to the research questions. Themes were classified into a concept matrix to facilitate comparisons. A concept matrix provides the transition from an author-to-concept-centric literature review, provides structure and helps in clarifying the concepts of the review for the reader.

The main themes identified were as follows:

- The design elements and interaction strategies used (addressing RQ1)
  ◦ the article's relevance to positive functioning
  ◦ how these elements and strategies support positive change
  - Input/output modalities (addressing RQ2)
    ◦ the type of XR
    ◦ the technology used
    ◦ the platform
    ◦ the outcome

These themes were based on the description provided in the articles, as crosschecked with other related and/or peer reviewed publication in the field to establish their scientific soundness, mainly toward nomenclature and interaction features. Next, the identified themes were normalized and classified so they would be easily comparable and fit into the concept spreadsheet in a valid and lossless way. Comparative studies that included two or more immersive, interactive experiences were tabulated in a respective number of rows.

In order to assess risk of bias (quality), we used the Downs and Black 26-item QAT scale (Downs and Black, 1998). A review article looked at 60 research evaluation systems and identified the Downs and Black checklist as one of the best evaluation systems available (Deeks et al., 2003). The Downs and Black checklist provides an overall quality index and four sub-scales of quality assessment: reporting, external quality, internal validity bias, and internal validity confounding. Answers are scored 0 or 1, except for one item that scored 0–2 making the maximum score possible 27. Generally speaking, scores are considered “excellent” (24–28 points), “good” (19–23 points), “fair” (14–18 points) or “poor” (< 14 points).

## Results

Of the 29 articles found in the scoping review process, some articles contained multiple systems and studies. Thus, we documented 33 immersive, interactive experiences relating to positive human functioning. However, we excluded 13 of those 33 XR experiences in the Downs and Black analysis because they were only proof of concept and did not have any participants, thus rendering the scale irrelevant. Therefore, we examined the remaining 20 experiences using the Downs and Black QAT scale. For the overall quality index, i.e., all 26 items comprising all sub-scales, a maximum score of 27 was possible. For the 20 experiences examined, the average overall quality index was 17.4 (SD = 2.96) with scores ranging from 12 to 23. Based on interpretations of this scale, these studies are considered fair to good with only one study performing poorly in terms of validity and reliability. One possible reason for the wide spread of scores is because the studies were for different audiences. For example, a psychology study might use similar metrics to the Downs and Black scale to assess quality and thus have a higher score compared to a user study or art installation that uses a different set of metrics to assess quality. Moreover, this metric was designed for medical intervention studies, which require a high degree of methodological quality; this is not necessarily the aim many of these articles we found here. Nonetheless, these results do show the range in methodological quality in the field and perhaps in the future researchers might consider using a similar metric to provide more rigor in their user study analyses.

### Design elements and interaction strategies

The 12 main themes that inform the design elements and interaction paradigms of the 33 documented immersive, interactive experiences are presented as follows.

- **Breath awareness**: Users' respiration data (inhale/exhale cycle) were recorded through either a respiration belt or microphone. These data were then employed in interaction design for users to become more mindful of their bodily processes (Davies and Harrison, 1996; Shaw et al., 2007; Hinterberger, 2011; Vidyarthi, 2012; Bal, 2013; Kitson et al., 2014; Prpa et al., 2015, 2016, 2017; Muñoz et al., 2016; Roo et al., 2016; Du Plessis, 2017) or achieve a relaxing state (van Rooij et al., 2016; Patibanda et al., 2017).
- **Concentration or focused attention:** Users' awareness of the present moment was supported through design that helps users bring their attention back when they have distracting thoughts. This was achieved explicitly through biofeedback (Shaw et al., 2007; Prpa et al., 2015, 2016; Amores et al., 2016; Kosunen et al., 2016; Muñoz et al., 2016) or implicitly by visual or auditory cues (Gu and Frasson, 2017; Navarro-Haro et al., 2017).
- **Connection:** Users can feel a sense of belonging and relatedness through telepresence and communication (Garau et al., 2003; Angelini et al., 2015; Sakamoto et al., 2015; Seaborn, 2016; Bernal and Maes, 2017; Quesnel and Riecke, 2017).
- **Emotional expression:** Emotions of the users can be expressed through audio and visual mappings, mainly through capturing physiological markers such as arousal (Bernal and Maes, 2017) and joy (Hinterberger, 2011).
- **Feedback of performance:** Users received some form of information about their performance. Feedback was given as virtual movement (Davies and Harrison, 1996; Amores et al., 2016; Kosunen et al., 2016; Du Plessis, 2017), change in visuals (Shaw et al., 2007; Hinterberger, 2011; Bal, 2013; Choo and May, 2014; Gromala et al., 2015; Prpa et al., 2015, 2017; Kosunen et al., 2016; Roo et al., 2016; van Rooij et al., 2016; Patibanda et al., 2017), or change in audio (Shaw et al., 2007; Hinterberger, 2011; Vidyarthi, 2012; Kitson et al., 2014; Prpa et al., 2015, 2016, 2017; Muñoz et al., 2016; Gu and Frasson, 2017).
- **Mind-body dialogues:** Users were able to explore the connection between their physical and mental states, the idea being that one similarly affects the other. A calm body breeds a calm mind: (Shaw et al., 2007; Bal, 2013; Gromala et al., 2015; Prpa et al., 2015; Kosunen et al., 2016; Muñoz et al., 2016; Roo et al., 2016; van Rooij et al., 2016; Du Plessis, 2017). To change ourselves, we need to change our perspectives: (Davies and Harrison, 1996). Color transmits and translates emotion (Wiethoff and Butz, 2010; Hinterberger, 2011). Music is the mediator between the spiritual and the sensual life: (Vidyarthi, 2012; Kitson et al., 2014; Prpa et al., 2016, 2017).
- **Mindfulness meditation:** These experiences involved paying attention on purpose, in the present moment, and nonjudgmentally. Users were guided through a narration (Shaw et al., 2007; Choo and May, 2014; Prpa et al., 2015; Gu and Frasson, 2017; Navarro-Haro et al., 2017) or had the chance to playfully discover meditation practice unguided (Davies and Harrison, 1996; Vidyarthi, 2012; Bal, 2013; Kitson et al., 2014; Gromala et al., 2015; Amores et al., 2016; Kosunen et al., 2016; Prpa et al., 2016; Roo et al., 2016; Du Plessis, 2017), while another experience incorporated but was not explicitly about mindfulness meditation (Chittaro et al., 2017).
- **Movement:** Users physically moved their bodies in order to interact with the system. Movement was used as a way to promote health (Eubanks, 2011; Seaborn, 2016) and also further immerse the user in the virtual space through embodiment (Davies and Harrison, 1996; Bal, 2013; Sakamoto et al., 2015; Quesnel and Riecke, 2017).
- **Nature elements:** These experiences involved some aspects of nature. Some experiences used water as a visualization (Bal, 2013; Sakamoto et al., 2015; van Rooij et al., 2016; Gu and Frasson, 2017; Prpa et al., 2017), while others used animals (Shaw et al., 2007; Eubanks, 2011; Sakamoto et al., 2015). A common theme was using park or garden elements (Choo and May, 2014; Angelini et al., 2015; Roo et al., 2016; Chittaro et al., 2017), while other experiences focused more specifically on trees and the forest (Davies and Harrison, 1996; Gromala et al., 2015; Patibanda et al., 2017). One experience used a sunset scenery (Shaw et al., 2007), and another used the entire Earth (Quesnel and Riecke, 2017).
- **Physiological measures:** Use of instruments that provide information on physiological functions in order to gain greater awareness of internal states of a user. The processes can include brainwaves (Hinterberger, 2011; Choo and May, 2014; Prpa et al., 2015, 2016; Amores et al., 2016; Kosunen et al., 2016; Du Plessis, 2017; Gu and Frasson, 2017), skin temperature and conductance (Shaw et al., 2007; Hinterberger, 2011; Gromala et al., 2015; Bernal and Maes, 2017; Du Plessis, 2017), respiration (Davies and Harrison, 1996; Shaw et al., 2007; Hinterberger, 2011; Vidyarthi, 2012; Bal, 2013; Kitson et al., 2014; Prpa et al., 2015, 2016, 2017; Roo et al., 2016; van Rooij et al., 2016; Du Plessis, 2017; Patibanda et al., 2017), and heart rate and heart rate variability (Shaw et al., 2007; Hinterberger, 2011; Muñoz et al., 2016; Roo et al., 2016; Bernal and Maes, 2017; Chittaro et al., 2017).
- **Playfulness:** Users were invited to interact with the system that supports curiosity and creativity in order to make the experience as inviting and non-invasive as possible. This was achieved through exploring a narrative (Eubanks, 2011; Amores et al., 2016; Muñoz et al., 2016), employing gaming mechanics (Choo and May, 2014; Sakamoto et al., 2015; Muñoz et al., 2016; Seaborn, 2016; van Rooij et al., 2016; Patibanda et al., 2017), and using active and imaginative elements (Wiethoff and Butz, 2010; Hinterberger, 2011; Vidyarthi, 2012; Kitson et al., 2014; Prpa et al., 2015, 2016, 2017; Roo et al., 2016).
- **Social presence:** Users interacted with other users at the same time (Angelini et al., 2015; Sakamoto et al., 2015; Seaborn, 2016; Bernal and Maes, 2017) or avatars that felt as if they were real people (Garau et al., 2003).

Physiological measures (*N* = 21), feedback loop (*N* = 19), and mind-body dialogues/mindfulness-meditation/play (all *N* = 16) were the design elements or interaction strategies most utilized. These results can inform the answer to RQ1.

### Input/output modalities

**Figure 3:**
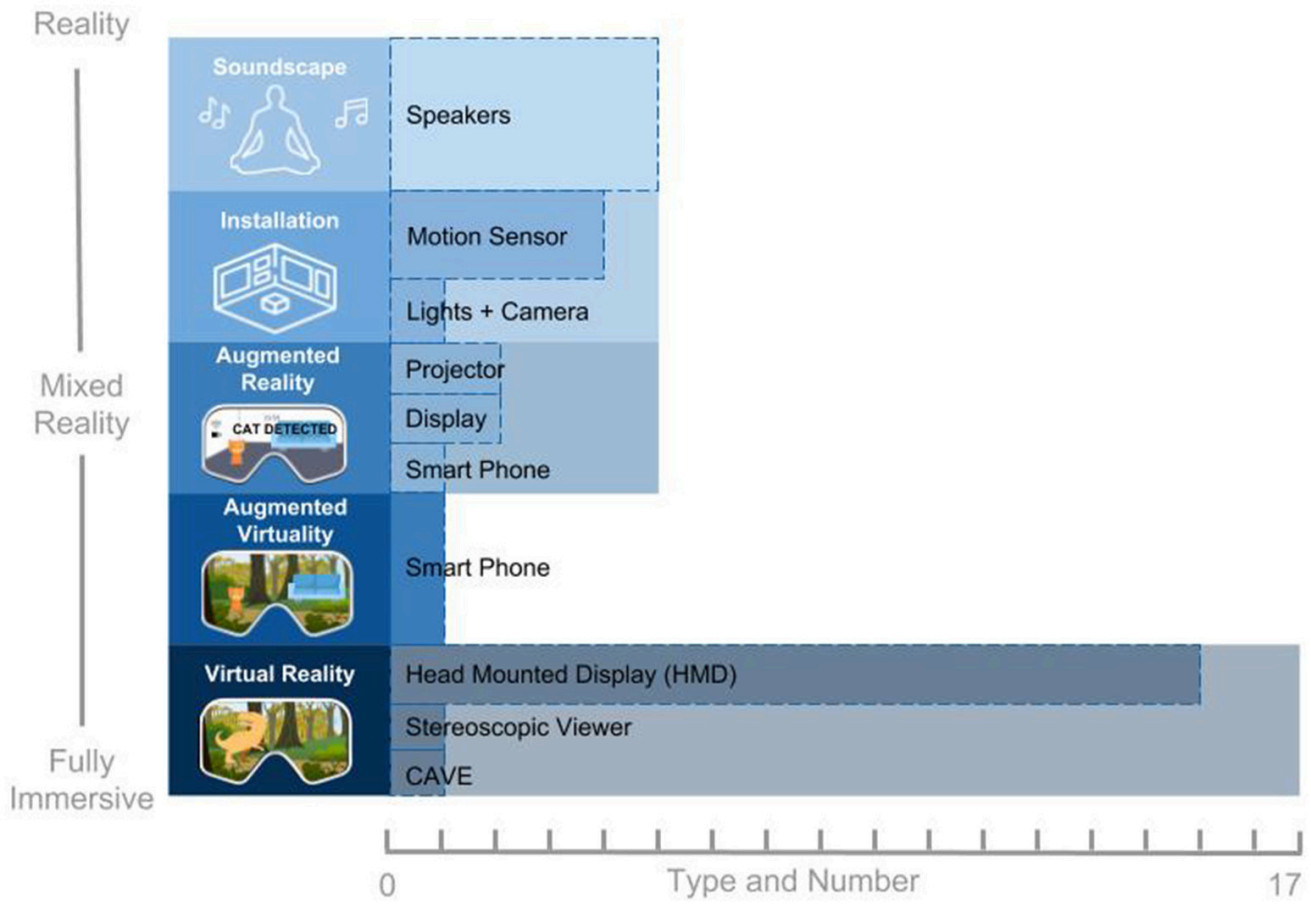
The type and number of immersive, interactive experiences for positive functioning (x-axis). Types of immersive, interactive experiences are categorized on a virtuality continuum (y-axis) that increases in immersive properties from soundscape (least immersed) to virtual reality (fully immersed). Each type is broken down into the kind and number of technology used, and this is represented as the dotted bars within the larger bars representing the total number of experiences.

**Figure 4:**
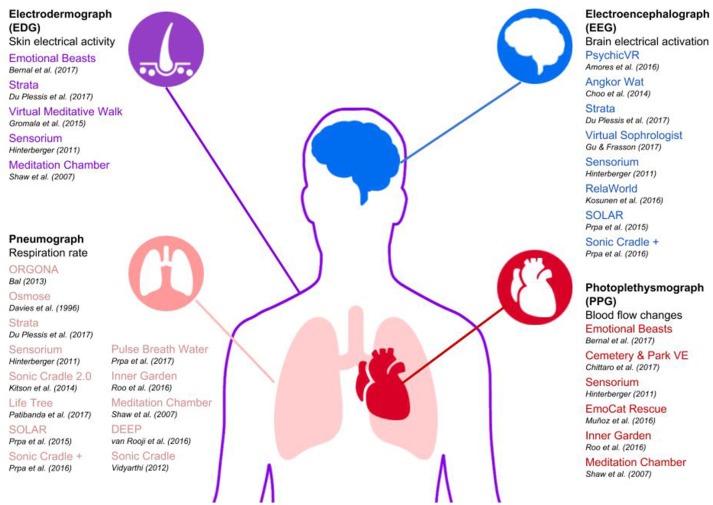
Biofeedback types and their corresponding immersive, interactive experiences. There are four types of biofeedback represented: electrodermograph (EDG), pneumograph (respiration rate), electroencephalograph (EEG), and photoplethysmograph (PPG). The names of the experience along with the author's name is listed. Some experiences are listed multiple times, indicating they used multiple types of biofeedback in their system.

**Figure 5:**
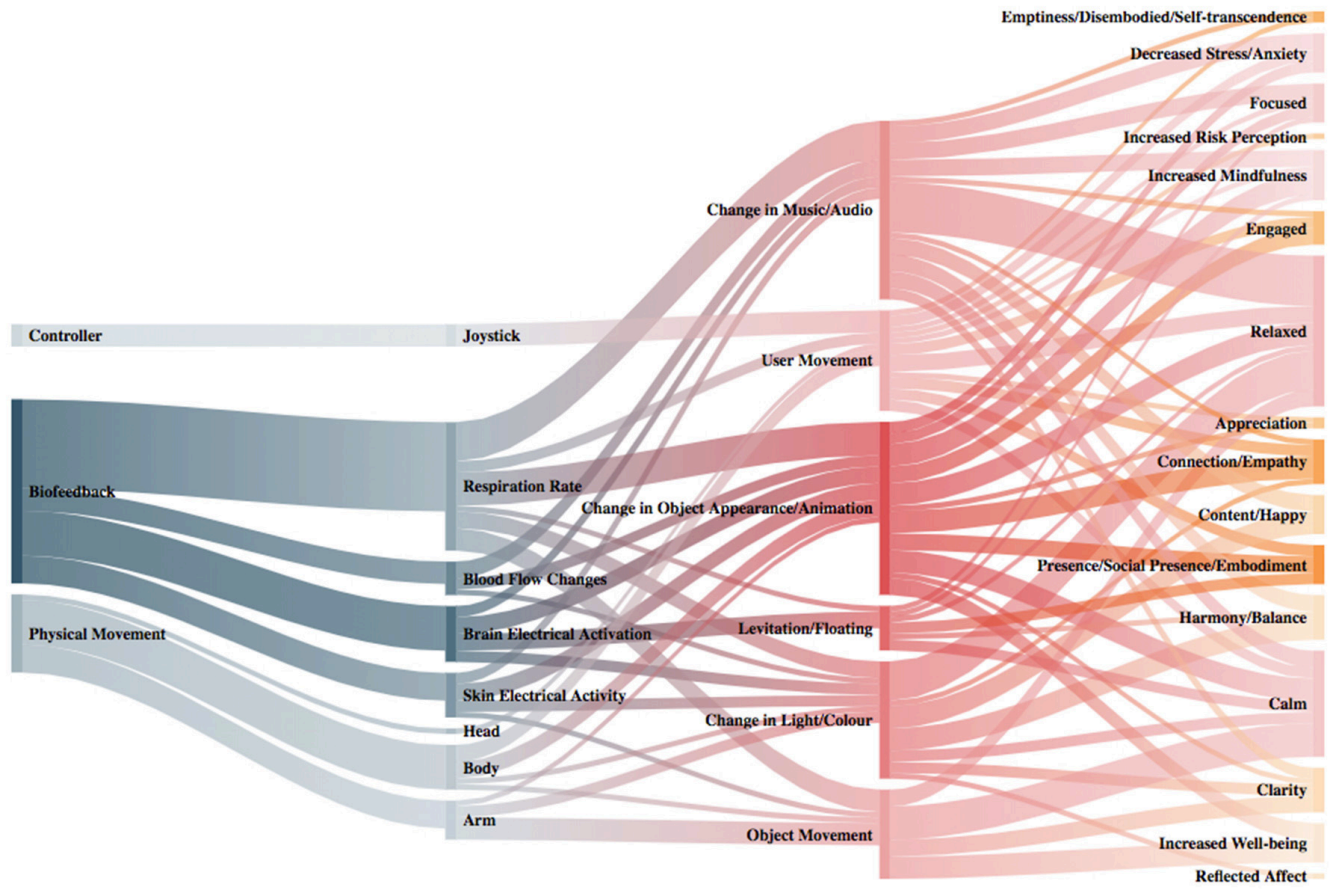
Sankey diagram showing the input-output modalities and their corresponding outcomes for all experiences. Please note that some experiences use multiple types of input-output modalities, and some inputs correspond to multiple outputs and outcomes. Color intensity and stroke breadth indicate number of experiences for that category going left to right. This figure represents a static image of the data. For an interactive diagram that shows the number of experiences for each category, please see https://akitson.github.io/.

To address RQ2, we extracted the input-output modalities of the experiences, the type of XR, the technology employed, and the platform used. The type of XR and technology employed can be seen in Figure 3. For a more detailed description of these data, we also created a table (see Supplementary Material) that shows both the technology and the platform used by each system individually, grouped by XR type. In terms of the input-output modalities, we grouped all the immersive, interactive positive experiences and categorized them into three high level themes: biofeedback, physical movement, and controller. Within each of these three high level themes were different input modalities. For biofeedback, this contained four types of inputs: blood flow changes, skin electrical activity, respiration rate, and brain electrical activation (see Figure 4). The physical movement theme contained three input types: arm, body, and head. The controller theme had two input types: joystick and screen. We then mapped these inputs to output modalities. We grouped the outputs into six different themes: change in music/audio, change in light/color, change in object appearance/animation, object movement, levitation/floating, and user movement. Finally, we mapped the six different types of outputs to 16 types of outcomes: relaxed, content/happy, reflected affect, increased mindfulness, harmony/balance, appreciation, calm, decreased stress/anxiety, connection/empathy, clarity, focus, increased well-being, emptiness/disembodied/self-transcendence, engaged, presence/social presence/embodied, and increased risk perception. A depiction of the input-output-outcome modalities can be found in Figure 5 and also accessed online here: https://akitson.github.io/.

## Discussion

Immersive interactive technologies have, so far, mainly been developed for applications such as entertainment and training. However, the potential for these technologies is vast and we are beginning to see the direction of the field shift toward more experiences of supporting positive human functioning and change (Fogg, 1999; Schiphorst, 2009; Sander, 2011; Riva et al., 2012; Brown, 2013; Desmet and Pohlmeyer, 2013; Kennedy, 2014; Buie, 2016; Gaggioli, 2016; Mossbridge, 2016). There are similar, yet separate, movements from different domains such as HCI, Psychology, and Computer Science all going toward this same goal of designing and creating technologies that support positive human functioning (Norman and Draper, 1986; Weiser and Brown, 1996; Seligman and Csikszentmihalyi, 2014). Yet, there is not a clear overview of all of these domains and what they have contributed so far. The diversity of the domains could be one reason there has not been a general XR for positive change review. In general, the current scoping review showed that the recent resurgence of XR technologies that are low-cost and accessible offered an opportunity to explore the medium further. Moreover, it enabled designers and technologists that ability to create more experiences, thus providing grounds for a comparison and analysis of the design elements and interaction strategies used, as well as the input-output-outcome modalities. Overall, the authors find this review shows promise for a new era of XR for positive change and that there exist enough experiences for researchers to map it and further develop significant conceptual knowledge for the research community and the public.

### Design elements and interaction strategies for supporting positive change in immersive, interactive technologies

We can make several observations from the reviewed and studied XR design elements and interaction strategies in section Design Elements and Interaction Strategies.We have organized the above 12 themes into four higher-level themes: instruments of analysis, phenomena and theoretical constructs, content features, and physical activity.

#### Instruments for analysis

First, **physiological measures** and **feedback of performance** are the most prevalent elements. There is considerable overlap between these two elements with all but one experience making use of physiological measures as a means to provide feedback on performance. Since its inception in the 1970s, biofeedback has been gaining popularity due to its use as a supporting mechanism that can offer explicit insights about the user's state and can guide a user to change their thoughts, emotions and behavior (Schwartz and Andrasik, 2017). However, biofeedback has been mainly used as a form of treatment in medicine and psychology and we have only recently seen more applications to immersive, interactive experiences; and this may be in part due to the dispersion of increasingly affordable and consumer-friendly physiological devices. The literature review also showed a preference for experiences using mind-body dialogues and mindfulness meditation interaction strategies. Both of these elements emphasize focusing on the body and noticing any sensations, thoughts or feelings that happen in the present moment (Kabat-Zinn, 2003). Studies have shown numerous benefits for mindfulness meditation such as reducing depression symptoms, stress, and anxiety (Chiesa and Serretti, 2010). Moreover, the same mindfulness processes understood by Buddhist traditions for many years have been brought to psychology and now to human-computer interaction design. Thus, it is perhaps not surprising that immersive, interactive technologies make use of these concepts to support positive change because they can provide a space one might not otherwise have access to explore their own internal bodily states. In fact, many experiences from the review also made use of two elements very closely related to mindfulness and mind-body dialogues: breath awareness and concentration or focused attention. Breath is often seen as an integral part of mindfulness meditation because it provides a focus point to bring one's attention back to the present moment when the mind wanders. Thus, bringing one's attention back to the breath, or some other focus of attention, works the mind and we gain more control over our internal states with each practice.

#### Phenomena and theoretical constructs

Another observation is that **emotional expression**, **connection**, and **social presence** are not studied or utilized as much as mindfulness meditation. One might expect more experiences with these elements given both that social integration and connectedness are important components in many psychological frameworks of well-being and positive human functioning (Ryff, 1989; Seligman, 2012; Venter, 2017), and that there is a movement in several domains to use technology as more than a distraction or consumption device and instead use it to connect with others as a part of health behavior change (Riva et al., 2012; Brown, 2013; Calvo and Peters, 2014; Kennedy, 2014; Mossbridge, 2016). Moreover, Höök has proposed the affective loop, where the system affects the user and the user affects the system (Höök, 2008). This represents a gap that can be addressed by future developments of immersive, interactive technologies for positive change.

#### Content features

**Nature** was another common design element in the immersive, interactive experiences we reviewed. Research evidence suggests that connecting with nature is one path to flourishing in life and positive mental health (for a review see Capaldi et al., 2015). We found similar benefits of enhanced mood, reduced stress, and increased well-being across the XR experiences that involved nature. Thus, it appears that the benefits of being in contact with nature can be replicated in a virtual or augmented environment. This is promising for using XR experiences to help support positive change for those who cannot have much access to nature or the outdoors, such as those in urban areas or in medical facilities.

#### Physical activity

Finally, about half of the experiences included in this review used the interaction strategies of **play** and **movement**. We can draw similarities between these elements and several existing theories: somaesthetics, the importance of the role of bodily experience in aesthetic appreciation (Schiphorst, 2009; Shusterman, 2012); embodied cognition, our mental constructs are shaped by aspects of the body (Varela et al., 1992; Markman and Brendl, 2005); play, in being creative we can reach across domains of meaning and forge new conceptual connections leaning to insight or cathartic release (Clark, 2013); and game play, gaming activities embody immense concentration, enjoyment, relationships, and accomplishment that can lead to improved mood, reduced emotional disturbance, improved emotion regulation, relaxation, and reduced stress (Jones et al., 2014). Future XR experiences aimed at supporting positive change would be strengthened by incorporating these theories from other disciplines because they have already demonstrated their effectiveness for supporting flourishing and positive mental health.

### Input-output mappings of immersive, interactive technologies for eliciting positive change

#### Immersive, interactive technologies

The review of technologies and platforms focused strongly on virtual reality (VR) technology. Therefore, it is perhaps unsurprising that VR, particularly the use of head-mounted displays (HMDs), is the most prevalent type of technology that we find compared to other mixed reality experiences. Immersive soundscapes are the second most common type of technology used for eliciting positive states. The use of other XR technologies along the mixed reality continuum of immersion seem to have been overlooked. One possible explanation could be that HMDs are being made increasingly more affordable and accessible, while also improving in overall quality; other mixed reality technologies are still in their infancy and lack the development support for designers to more easily create experiences. The authors would like to emphasize that simply because VR is currently the most prevalent technology used in eliciting positive states does not necessarily mean it is the best platform. Each design requires careful consideration of the intended experience and specific outcomes when selecting a platform, taking into account the context and its users, and more research is needed for determining the “best” platform for eliciting positive change.

#### Input

The review of input-output modalities showed that physiological data was the most predominant type of input, followed by physical movement and then controller (see Figure 5). When breaking down the type of biofeedback used, we found that respiration rate was overwhelmingly the most utilized type (*N* = 16). Measuring respiration rate is relatively non-invasive and the data is reliable compared to the other types of biofeedback such as EEG; this may partially account for its high use. As was discussed previously, breath is an important component in mindfulness meditation and a reliable way to decrease stress. Therefore, using respiration rate as an input is congruent with the mindfulness and mind-body dialogue interaction strategies used in these experiences for positive change.

One observation we made about the type of input is that there was a low number of experiences using controllers, such as joysticks or touch screens. This might be surprising considering that much of the XR industry is being fueled by entertainment and gaming applications that make use of traditional controller-based inputs. This review perhaps demonstrates that traditional controllers do not map well to eliciting positive states. We hypothesize this is due to controllers' arduous nature that might lead to a break in presence, immersion and flow, and subsequently distracting from the goal of eliciting positive states. However, further research is needed.

Physiological input was very prevalent in the studies and experiences we reviewed, with 34 instances of mappings involving physiological input. And, although there are many benefits to using physiological measures such as getting a more empirical measures of users' inner states, there are also several shortcomings that we would like to highlight in this review for designers and research hoping to use physiological measures in their XR experiences. First, there can be considerable noise in the data, especially EEG measures of brain electrical activation (Ramirez and Vamvakousis, 2012). Moreover, wearing physiological sensors might feel cumbersome to the user, which may distract from the desired user experience.

#### Output

Change in object appearance/animation was by far the most common type of output (*N* = 18), compared to change in music/audio (*N* = 14), change in light/color (*N* = 11), object movement (*N* = 10), user movement (*N* = 10), and levitation/floating (*N* = 4). Changes in music/audio and changes in object appearance/animation were more likely to be matched with respiration and relaxation or calm, whereas object and user movement were more likely to be matched with engagement and clarity. These outputs are in keeping with the literature: breath meditation can lead to relaxation and calmness (Carter and Carter, 2016), and physical activities can bring about engagement and positive health outcomes (Gao et al., 2015). The current state of the XR technology is primarily focused on visuals, so it is not surprising to find most experiences using this in their interactivity. Audio and music are also easily modified through speakers and headphones. One observation is that some of the other human senses are underutilized, such as smell, touch, and temperature. Some experiences make use of tangibles (Angelini et al., 2015; Sakamoto et al., 2015; Roo et al., 2016), but there is still a lot of work to be done in going outside visuals and audio for XR interactivity. In terms of well-being, emotion and memory are closely linked with the olfaction; odors that evoke positive autobiographical memories have the potential to increase positive emotions, decrease negative mood states, disrupt cravings, and reduce physiological indices of stress, including systemic markers of inflammation (Herz, 2016).

#### Outcome

Finally, the outcomes of using respiration rate as an input were relaxation, calmness, increased well-being, and decreased stress/anxiety. From these results, it appears that the main mechanism for eliciting positive states is through using biofeedback that is mapped to some kind of change in sensation in the XR environment, whether that be a change in music/audio, light/color, or object appearance/animation; this feedback of physiological performance then allows users to experience an internal state from a different perspective and thus start to form the ability to change that state. It appears that practicing an awareness and control of one's internal physiological states can lead to positive states such as relaxation, calmness, harmony/balance, clarity, focus, and increased well-being. From this mapping we saw that the outcomes were calmness, contentment/happiness, presence/embodiment, and engagement. Thus, the physical and virtual movement connection seems to have contributed to eliciting positive states. And, when we look at the interaction strategies employed for these systems, we see play and movement are important. This is, the sense of curiosity, imagination, and embodiment in these experiences are all common themes and elements that allow the user to explore a system in a more natural and familiar way than a more abstract way of interacting like the traditional joystick. This idea of natural interaction supporting the desired user experience of curiosity, imagination, and embodiment in XR is maintained by several studies (Beckhaus et al., 2005; Macaranas et al., 2015; Desai et al., 2016; Quesnel and Riecke, 2017).

### A framework for immersive interactive technologies for positive change

**Figure 6:**
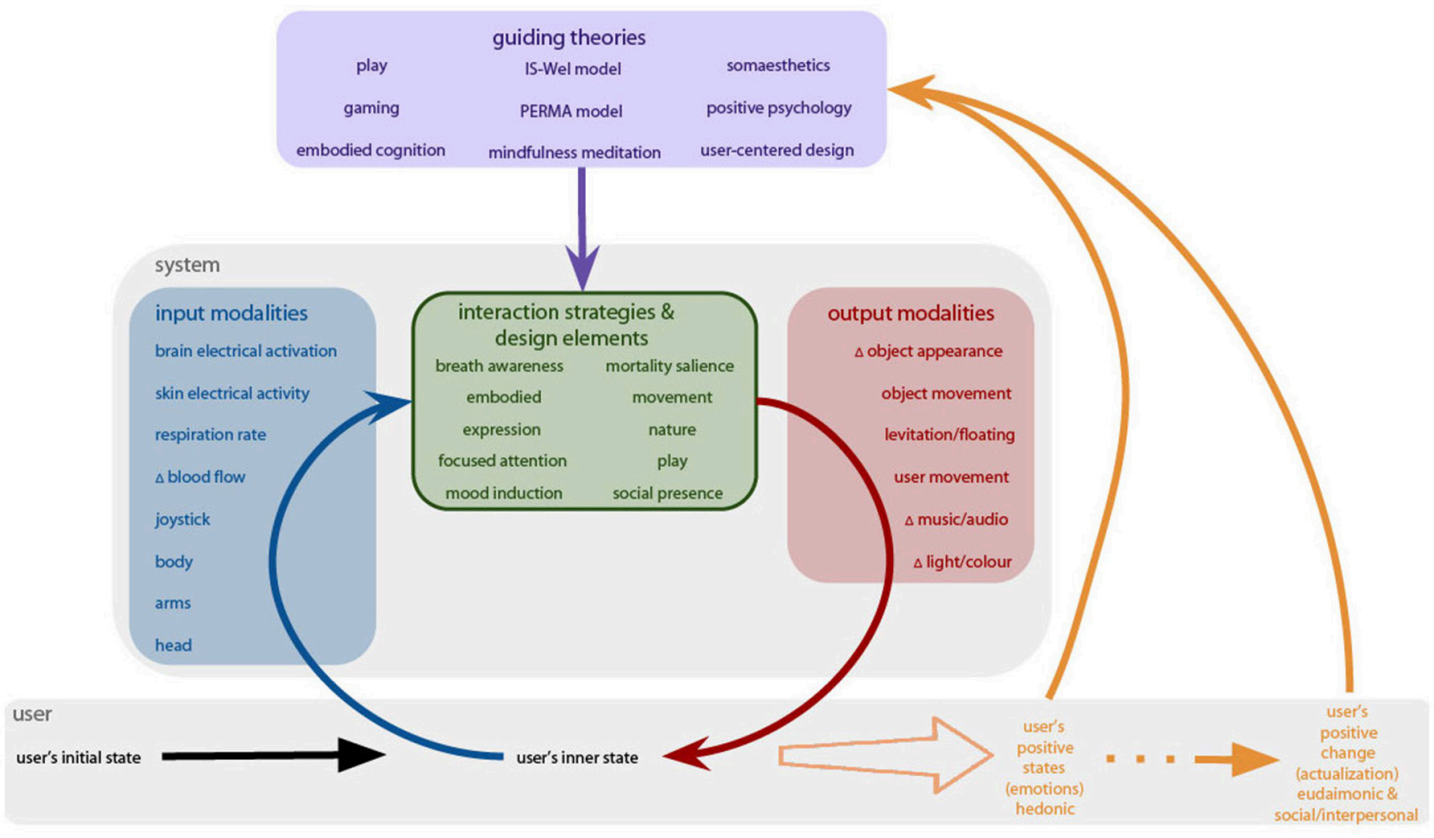
A schematic of the framework for designing immersive, interactive experiences for eliciting positive states and supporting positive change.

Several frameworks have already been proposed for designing technologies for eliciting positive human functioning and well-being: Anthropology-Based Computing (Brown, 2013); Techno-spiritual Design (Buie, 2016); Positive Computing (Sander, 2011; Calvo and Peters, 2014); Positive Design (Desmet and Pohlmeyer, 2013); Persuasive Technology (Fogg, 1999); Computer-mediated Self-transcendence (Gaggioli, 2016); Technowellness (Kennedy, 2014); Transcendence Technology (Mossbridge, 2016); User-centered Design (Norman and Draper, 1986); Positive Technology (Riva et al., 2012); Calm Technology (Weiser and Brown, 1996); Affective Computing (Picard, 1995); Ergonomics (Jastrzebowski, 1857; Edholm and Murrell, 1973); Hedonomics (Helander, 2002); Value-Sensitive Design (Friedman and Kahn, 1992); Emotional Design (Norman, 2004)—see also Figure 1. However, these frameworks do not focus on immersive, interactive technologies (XR) in particular. Therefore, we offer a more focused and concrete framework for designing immersive, interactive technologies for eliciting positive states and supporting positive change (see Figure 6). This framework is constructed from the results of this scoping review: the interaction strategies and design elements, the input-output modalities that incorporate the use of XR technology, and the outcomes that resulted from the user's interaction with the system.

The designer or researcher has positive state(s) or positive change in mind as the outcome (orange). These outcomes will influence the theories and models considered when designing the experience (purple). Those theories in turn will help to inform the interaction strategies and design elements used (green). And, the interaction and design elements will then inform the feedback loop of input (blue) and output (red) modalities. Therefore, when the user is put into the system, their inner state is measured and collected via physiological measures and movement data. These data of the user's inner states are then fed into the system and represented/externalized in an abstracted way as the output modality. The user then experiences their own inner state that can change their initial state is then fed back into the system. Thus, the system and the user influence one another. This feedback loop over time can build positive experiences and contribute to a positive state. Eventually, this feedback loop shapes positive states, which then might lead to positive change in the user.

### Design considerations for future immersive interactive technologies for positive change

In addition to the themes listed and discussed above, which might be useful as descriptive tools for researchers, we now present a set of prescriptive design considerations to serve as tools for designers and developers interested in creating immersive, interactive systems, and experiences with the goal of eliciting positive states and supporting positive change. We want to note, however, that no formula exists to make someone have a given experience. We can only submit our best practices for giving the user suitable conditions and opportunities for them to engage if they wish.

#### Consider the outcome and human experience first, then work backwards

The intention or goal behind your work will shape every design decision, so it is important to have a clear sense of what specific positive state or positive change you wish to support with the XR technology. Trying to force the user to accommodate a technology that is not in keeping with their natural way of interacting and experiencing the world, even if it is a virtual one, can lead to frustration, negative emotions, and disconnect; this is in keeping with user-centered design and the existing frameworks for supporting positive functioning through technology (Norman and Draper, 1986; Fogg, 1999; Riva et al., 2012; Calvo and Peters, 2014).

#### Consider using sensory changes to support relaxation, contentment, and harmony/balance

Our results of the scoping review for the input-output modalities (RQ2) suggest that specific changes in either music/audio, object appearance/animation, or light/color can be associated with outcomes of relaxation, contentment, and harmony. These positive states are more subdued in feeling; therefore, the changes in the virtual environment too are subtle yet obvious enough to the user that there is in fact a change occurring. Hinterberger (2011) uses changes in light and sound to achieve all three of these positive states, while both Shaw et al. (2007) and Gromala et al. (2015) use changing imagery of jellyfish and fog, respectfully, to support relaxation. These sounds, animations, and colors used in the XR experiences all seem to support their desired outcome in some way, whether that be red colors for engagement, soft music with a low tempo or a setting sun for relaxation.

#### Consider using movement to support calmness, clarity, and focus

Results showed that movement of any kind, i.e., user movement and object movement, was linked to positive states of calmness, clarity, and focus. More specifically, big sweeping physical movements of the user, and expanding/contracting of virtual objects in rhythm with the user's input helped to support positive states of calmness, clarity, and focus. This result is perhaps due to a release of bodily tension and stress, though more research is needed. These positive states are more active than the ones mentioned above because the user is physically engaged in the experience. Bal's (2013) ORGONA project serves as a good example of using physical movement to support these three positive states because the user engages their body and focuses on their breathing to move virtual objects. Another good example is Muñoz et al. (2016) EmoCat Rescue game where users must focus on controlling their breathing and heart rate in order to progress in the game.

#### Consider using biofeedback for mediating changes to the virtual environment

From the review, we found that physiological data was most commonly mapped directly to changes in the system, whether that be changes in music/audio, light/color, or object appearance/animation. Users reported feedback that allows them to externalize and notice their internal states in the virtual environment helped them to better understand their own internal states, and maybe even gain more control of them (Vidyarthi, 2012; Patibanda et al., 2017). Our finding is supported by other research that shows biofeedback is effective in interactive technologies aimed at improving mindfulness (for a review see Sliwinski et al., 2017). Moreover, the design considerations from Patibanda et al. (2017) provide positive evidence for using respiration rate as a form of biofeedback in games: use subtle onboarding, use non-interruptive breathing feedback, provide imitative breathing feedback, use a minimalist approach to designing naturalistic visuals, and use hardware that considers breathing performance and increases self-awareness of breathing. Other forms of biofeedback we found in the review include blood flow changes, skin electrical activity, and brain electrical activation. While there are less examples of concrete experiences for these biofeedback elements, we can still observe that the majority of mappings for both blood flow changes and skin electrical activity are to more subtle changes in music/audio and light/color, whereas brain electrical activation is primarily mapped to more obvious changes in object appearance/animation and levitation/floating. One reason for this might be that it is less obvious to the user when their brain state is changing rather than a change in heart rate or sweating, which we can physically feel or see more directly. Therefore, we suggest using a reverse proportional mapping—the harder it is to notice a physiological change, the more obvious the feedback should be in the virtual environment, and vice versa.

#### Consider mapping physical and virtual movement together

We observed that the use of physical movement and controller interaction strategies were most often mapped to corresponding virtual object or user movement. The use of physical movement in a virtual environment is important because it allows the user to feel more immersed in the experience. One study examined how users experience movements in their interaction with interactive systems and identified four features of movement-based interaction that potentially influence immersion: natural control, mimicry of movements, proprioceptive feedback, and physical challenge (Pasch et al., 2009). The models of immersion in this study were based off of two theories: Csikszentmihalyi's (1990) Flow theory, a state of optimal experience where people typically have deep enjoyment, creativity, and total involvement in life; and Brown and Cairn's (2004) immersion framework of engagement, engrossment, and total immersion. Thus, physical movement and locomotion in immersive interactive experiences might help support positive states and change, especially if we are to follow the guidelines mentioned above put forth by Pasch et al. (2009), as well as maintain immersion and user experience.

#### Consider natural elements, minimalist design, and child-like play for design elements and interaction strategies

Many theoretical papers have already proposed using natural elements, minimalist design, and child-like play in interaction design (Schultz and Tabanico, 2007; Vidyarthi and Riecke, 2014; Capaldi et al., 2015; Ahn et al., 2016; Gaggioli, 2016). And, indeed, we found this to be true in the experiences we reviewed. Several studies we reviewed also found that using nature elements in the virtual environment (*N* = 15), taking minimalist approach (*N* = 7), and adopting a child-like play concept for interaction design (*N* = 16) all contributed to positive states or positive change in users—see results section for details on the specific studies. The use of abstract imagery in particular for taking a minimalist approach seemed to help users focus their attention and block out any external distractions; this abstract imagery also helps users to focus on something that does not come with preconceived ideas or feelings that may trigger an unwanted emotional response.

#### Consider the type of technology last based on your desired goals and user experience

Finally, the type of technology used should be the last thing a designer should consider for their XR experience if they are to be in keeping with the principles of user-centered design. More explicitly, the technology or platform selected should support and enhance the desired user experience and outcomes. The goal should not be to use a certain technology simply because it is “cutting edge.” We are seeing more and more XR technologies emerging, and that is promising for the field. However, the authors caution XR designers to think through why they are using a certain technology, and might another technology be a better fit? It should be clear how the XR technology elicits positive states and supports positive change, as well as how the extra effort of using XR technology is justified. The experiences we have seen so far, from this review, show that many are using virtual reality and in particular head-mounted displays. While this platform is great for total immersion, there still exist other forms of XR that might be equally or more beneficial; more research and development of experiences for other XR types is needed.

### Limitations

The diverse nature of the various XR experiences and their accompanying studies presented challenges, leading to a series of compromises and assumptions that could be perceived as limitations in the literature review.

First, an XR experience can integrate two or more interaction strategies and input-output modalities to support positive change. For example, pulse, brain potential shifts, and skin conductance can all influence the virtual environment's visuals and audio in different ways (Hinterberger, 2011). These kinds of integrations include a dominant outcome. In this review, the XR components were analyzed based on their dominant outcomes. For example, in the example above the outcomes were contentment, relaxation, happiness, and harmony. However, the distinction of what elements contributed to which specific outcome could not be determined from this review and so were considered together.

A second limitation is the vast differences in using empirical methods in all the studies identified for this review. Several of the studies included were only proof of concept (Choo and May, 2014; Sakamoto et al., 2015; Muñoz et al., 2016; Bernal and Maes, 2017; Du Plessis, 2017); Thus we cannot determine for sure that these interaction techniques will reliably elicit those same outcomes.

Another limitation is in the generalizability of the reported outcomes because many studies used university students as participants. It is unclear whether the same outcomes will hold for the general population or more vulnerable populations.

Finally, the database query of the review is based on a predefined set of search terms. The defined search strategy conforms to the established procedures for scoping literature reviews, breaking down and addressing the research questions while ensuring reproducibility of the search. Yet, XR is a dynamic and vast field covering many different research fields; all of these fields have different terminologies and search terms that make it challenging to uncover every XR work that relates to positive states and change. For related reviews on neighboring topics see these works: Plaza et al. (2013), Capaldi et al. (2015), Mossbridge (2016), Spanakis et al. (2016), Valmaggia et al. (2016), and Sliwinski et al. (2017). Future scoping or systematic reviews on the topic might include the following terms, which are based on the key terms from the included literature in this review: virtuality, cinematic reality, computer-mediated reality, alternate reality, wearable computing, visuo-haptic mixed reality, games for health, HCI for peace, value-sensitive design, biofeedback, emotional design, holistic health, mediated communication, physiological computing, interactive art, multisensory experience, self-expression, prosocial behavior, cultural worldview, narrative exercises, mood-induction procedures, and self-regulation.

## Conclusions

We presented a scoping literature review of existing immersive, interactive technologies whose primary aim is to elicit positive states or support positive functioning. We discovered several ways to most effectively employ different design elements and interaction strategies to support positive change in users, as well as how to use input-output modalities to contribute to eliciting positive states. From this review, we formed a conceptual framework that may help researchers and designers think about immersive, interactive experiences in the context of positive states and positive change. In order to put forth a more concrete strategy for designers and creators to use this knowledge, we also provided a set of design considerations that also build on existing literature. The work presented here provides both researches and designers with a more organized and coherent sense of the existing literature on the subject across multiple fields.

Future work might address empirical evidence of how immersive, interactive experiences can elicit positive states or support positive change as this was something we found lacking in the literature. Another potential gap for designers to address is the creation of immersive, interactive experiences for social/inter personal outcomes, opposed to hedonic and eudaimonic outcomes that we found to be a lot more prevalent.

## Author contributions

AK, MP, and BR contributed conception and planning of the scoping review. AK and MP formulated the inclusion/exclusion criteria and identified articles relevant to the topic. AK performed the screening and eligibility process, and conducted a qualitative research synthesis of the data. AK wrote the first draft of the manuscript. All authors contributed to manuscript revision, read and approved the submitted version.

### Conflict of interest statement

The authors declare that the research was conducted in the absence of any commercial or financial relationships that could be construed as a potential conflict of interest.

## References

[B1] Ahn S. J. (Grace) Bostick J. Ogle E. Nowak K. L. McGillicuddy K. T. Bailenson J. N. (2016). Experiencing nature: embodying animals in immersive virtual environments increases inclusion of nature in self and involvement with nature. J. Comput. Mediat. Commun. 21, 399–419. 10.1111/jcc4.12173

[B2] Amores J. Benavides X. Maes P. (2016). PsychicVR: increasing mindfulness by using virtual reality and brain computer interfaces, in Proceedings of the 2016 CHI Conference Extended Abstracts on Human Factors in Computing Systems CHI EA'16 (New York, NY: ACM), 2–2.

[B3] Angelini L. Caon M. Couture N. Khaled O. A. Mugellini E. (2015). The multisensory interactive window: immersive experiences for the elderly, in Adjunct Proceedings of the 2015 ACM International Joint Conference on Pervasive and Ubiquitous Computing and Proceedings of the 2015 ACM International Symposium on Wearable Computers UbiComp/ISWC'15 Adjunct (New York, NY: ACM), 963–968.

[B4] Arksey H. O'Malley L. (2005). Scoping studies: towards a methodological framework. Int. J. Soc. Res. Methodol. 8, 19–32. 10.1080/1364557032000119616

[B5] Armstrong R. Hall B. J. Doyle J. Waters E. (2011). ‘Scoping the scope' of a cochrane review. J. Public Health 33, 147–150. 10.1093/pubmed/fdr015 21345890

[B6] Bal H. (2013). Responsive Aesthetics for Yogic Meditation: an Innovative Design Theory for Holistic Health That Supports Autonomy and Effective Training. Masters thesis, OCAD University.

[B7] Bazin A. (1967). What is Cinema? Vol. I Transl. by Gray H. Berkeley, CA: University of California.

[B8] Beckhaus S. Blom K. J. Haringer M. (2005). Intuitive, hands-free travel interfaces for virtual environments, in New Directions in 3D User Interfaces Workshop of IEEE VR (Bonn), 57–60.

[B9] Bernal G. Maes P. (2017). Emotional beasts: visually expressing emotions through avatars in VR, in Proceedings of the 2016 CHI Conference Extended Abstracts on Human Factors in Computing Systems CHI EA'17 (New York, NY: ACM), 2395–2402.

[B10] Bjork S. Holopainen J. (2005). Patterns in Game Design (Game Development Series). Charles River Media.

[B11] Botella C. Banos R. M. Guillen V. (2017). Positive technologies for improving health and well-being, in Positive Psychology Interventions in Practice, ed Proctor C. (Cham: Springer International Publishing), 219–234.

[B12] Bowman D. A. McMahan R. P. (2007). Virtual reality: how much immersion is enough? Computer 40, 36–43. 10.1109/MC.2007.257

[B13] Brown E. Cairn's P. (2004). A grounded investigation of game immersion, in CHI'04 Extended Abstracts on Human Factors in Computing Systems CHI EA'04 (New York, NY: ACM), 1297–1300.

[B14] Brown J. N. A. (2013). It's as easy as ABC introducing anthropology-based computing, in Advances in Computational Intelligence, Pt I, eds Rojas I. Joya G. Gabestany J. (Berlin: Springer-Verlag Berlin), 1–16.

[B15] Buie E. (2016). Transcendhance: a game to facilitate techno-spiritual design, in Proceedings of the 2016 CHI Conference Extended Abstracts on Human Factors in Computing Systems CHI EA'16 (New York, NY: ACM), 1367–1374.

[B16] Calvo R. A. Peters D. (2014). Positive Computing: Technology for Wellbeing and Human Potential. Cambridge, MA: MIT Press.

[B17] Capaldi C. A. Passmore H.-A. Nisbet E. K. Zelenski J. M. Dopko R. L. (2015). Flourishing in nature: a review of the benefits of connecting with nature and its application as a wellbeing intervention. Int. J. Wellbeing 5, 1–16. 10.5502/ijw.v5i4.1

[B18] Carter K. S. Carter R. III. (2016). Breath-based meditation: a mechanism to restore the physiological and cognitive reserves for optimal human performance. World J. Clin. Cases 4, 99–102. 10.12998/wjcc.v4.i4.99 27099859 PMC4832119

[B19] Chiesa A. Serretti A. (2010). A systematic review of neurobiological and clinical features of mindfulness meditations. Psychol. Med. 40, 1239–1252. 10.1017/S0033291709991747 19941676

[B20] Chittaro L. Sioni R. Crescentini C. Fabbro F. (2017). Mortality salience in virtual reality experiences and its effects on users' attitudes towards risk. Int. J. Hum. Comput. Stud. 101, 10–22. 10.1016/j.ijhcs.2017.01.002

[B21] Choo A. May A. (2014). Virtual mindfulness meditation virtual reality and electroencephalography for health gamification, in 2014 IEEE Games Media Entertainment (Toronto, ON), 1–3.

[B22] Clark C. (2013). The state of play. Int. J. Play 2, 161–162. 10.1080/21594937.2013.853462

[B23] Coelho C. Tichon J. G. Hine T. J. Wallis G. M. Riva G. (2006). Media presence and inner presence: the sense of presence in virtual reality technologies, in From Communication to Presence: Cognition, Emotions and Culture towards the Ultimate Communicative Experience. Festschrift in honor of Luigi Anolli, eds Riva G. Anguera M. T. Wiederhold B. K. Mantovani F. (Amsterdam: IOS Press), 25–45.

[B24] Cohen J. T. Neumann P. J. Weinstein M. C. (2008). Does preventive care save money? Health Econ. Pres. Cand. N. Engl. J. Med. 358, 661–663. 10.1056/NEJMp0708558 18272889

[B25] Csikszentmihalyi M. (1990). Flow: The Psychology of Optimal Experience. New York, NY: Harper Perennial Modern Classics.

[B26] Cummings J. J. Bailenson J. N. (2016). How immersive is enough? A meta-analysis of the effect of immersive technology on user presence. Media Psychol. 19, 272–309. 10.1080/15213269.2015.1015740

[B27] Davies C. Harrison J. (1996). Osmose: towards broadening the aesthetics of virtual reality. SIGGRAPH Comput. Graph 30, 25–28. 10.1145/240806.240808

[B28] Deeks J. J. Dinnes J. D'Amico R. Sowden A. J. Sakarovitch C. Song F. (2003). Evaluating non-randomised intervention studies. Health Technol. Assess. 7, iii–x, 1–173. 10.3310/hta7270 14499048

[B29] Desai S. Blackler A. Popovic V. (2016). Intuitive interaction in a mixed reality system, in School of Design; Creative Industries Faculty (Brighton).

[B30] Desmet P. M. A. Pohlmeyer A. E. (2013). Positive design: an introduction to design for subjective well-being. Int. J. Des. 7, 5–19. Available online at: http://www.ijdesign.org/index.php/IJDesign/article/view/1666/595

[B31] Diemer J. Alpers G. W. Peperkorn H. M. Shiban Y. Mühlberger A. (2015). The impact of perception and presence on emotional reactions: a review of research in virtual reality. Front. Psychol. 6:26. 10.3389/fpsyg.2015.00026 25688218 PMC4311610

[B32] Downs S. H. Black N. (1998). The feasibility of creating a checklist for the assessment of the methodological quality both of randomised and non-randomised studies of health care interventions. J. Epidemiol. Commun. Health 52, 377–384. 10.1136/jech.52.6.377 9764259 PMC1756728

[B33] Du Plessis I. (2017). Strata: a biometric VR experience, in ACM SIGGRAPH 2017 VR Village SIGGRAPH'17 (New York, NY: ACM), 14:1–14:2.

[B34] Edholm O. G. Murrell K. F. H. (1973). The Ergonomics Research Society: A History 1949-1970. Ergon. Soc. Publ. London: Taylor and Francis.

[B35] Ermi L. Mäyrä F. (2005). Fundamental components of the gameplay experience: analysing immersion, in Worlds in Play: International Perspectives on Digital Games Research, eds de Castell S. Jenson J. (New York, NY: Peter Lang), 37–53.

[B36] Eubanks A. (2011). Catching fireflies: a persuasive augmented reality game for android phones, in Proceedings of the 49th Annual Southeast Regional Conference ACMSE 11 (Kennesaw, GA), 363–364.

[B37] Fogg B. J. (1999). Persuasive Technologies. Comm. ACM 42, 27–29.

[B38] Fredrickson B. L. (2001). The role of positive emotions in positive psychology: the broaden-and-build theory of positive emotions. Am. Psychol. 56, 218–226. 10.1037/0003-066X.56.3.218 11315248 PMC3122271

[B39] Friedman B. Kahn P. H. (1992). Human agency and responsible computing implications for computer system design. J. Syst. Softw. 17, 7–14.

[B40] Friedman B. Kahn P. H. Borning A. (2006). Value sensitive design and information systems, in Human-Computer Interaction in Management Information Systems (Dordrecht: Springer), 55–95.

[B41] Gaggioli A. (ed.). (2016). Transformative experience design, in Human Computer Confluence, Transforming Human Experience Through Symbiotic Technologies (De Gruyter; Sciendo), 96–121.

[B42] Gaggioli A. Chirico A. Triberti S. Riva G. (2016). Transformative interactions: designing positive technologies to foster self-transcendence and meaning, in CyberPsychology, CyberTherapy and Social Networking Conference (CYPSY21) (Dun Laoghaire), 169–175.

[B43] Gaggioli A. Riva G. Peters D. Calvo R. A. (2017). Positive technology, computing, and design: shaping a future in which technology promotes psychological well-being, in Emotions and Affect in Human Factors and Human-Computer Interaction, ed Jeon M. (Cambridge, MA: Elsevier), 477–502.

[B44] Gao Z. Chen S. Pasco D. Pope Z. (2015). A meta-analysis of active video games on health outcomes among children and adolescents. Obes. Rev. 16, 783–794. 10.1111/obr.12287 25943852

[B45] Garau M. Slater M. Vinayagamoorthy V. Brogni A. Steed A. Sasse M. A. (2003). The impact of avatar realism and eye gaze control on perceived quality of communication in a shared immersive virtual environment, in Proceedings of the SIGCHI Conference on Human Factors in Computing Systems CHI'03 (New York, NY: ACM), 529–536.

[B46] Grau O. (2003). Virtual Art: From Illusion to Immersion. Cambridge, MA: MIT Press.

[B47] Grimshaw M. (2013). The Oxford Handbook of Virtuality. Oxford, UK: Oxford University Press.

[B48] Gromala D. Tong X. Choo A. Karamnejad M. Shaw C. D. (2015). The virtual meditative walk: virtual reality therapy for chronic pain management, in Proceedings of the 33rd Annual ACM Conference on Human Factors in Computing Systems CHI'15 (New York, NY: ACM), 521–524.

[B49] Gu G. Frasson C. (2017). Virtual sophrologist: a virtual reality neurofeedback relaxation training system, in Brain Function Assessment in Learning Lecture Notes in Computer Science, eds Frasson C. Kostopoulos G. (Cham: Springer), 176–185. 10.1007/978-3-319-67615-9_16

[B50] Hancock P. A. Pepe A. A. Murphy L. L. (2005). Hedonomics: the power of positive and pleasurable ergonomics. Ergon. Des. Q. Hum. Factors Appl. 13, 8–14. 10.1177/106480460501300104

[B51] Helander M. G. (2002). Hedonomics - affective human factors design. Proc. Hum. Factors Ergon. Soc. Annu. Meet. 46, 978–982. 10.1177/15419312020460120914612318

[B52] Herz R. S. (2016). The role of odor-evoked memory in psychological and physiological health. Brain Sci. 6:22. 10.3390/brainsci6030022 27447673 PMC5039451

[B53] Hinterberger T. (2011). The sensorium: a multimodal neurofeedback environment. Adv. Hum. Comp. Int. 2011:724204 10.1155/2011/724204

[B54] Höök K. (2008). Affective loop experiences – what are they?, in Persuasive Technology Lecture Notes in Computer Science, eds Oinas-Kukkonen H. Hasle P. Harjumaa M. Segerståhl K. Øhrstrøm P. (Berlin; Heidelberg: Springer), 1–12.

[B55] Höök K. Ståhl A. Jonsson M. Mercurio J. Karlsson A. Johnson E.-C. B. (2015). Cover story somaesthetic design. Interactions 22, 26–33. 10.1145/2770888

[B56] IJsselsteijn W. Riva G. (2003). Being there: the experience of presence in mediated environments, in Being There: Concepts, Effects and Measurements Of User Presence In Synthetic Environments Studies in New technologies and Practices in Communication, eds Riva G. Davide F. Ijsselsteijn W. (Amsterdam: IOS Press), 3–16.

[B57] Jastrzebowski W. B. (1857). An Outline of Ergonomics, or the Science of Work Based Upon the Truths Drawn From the Science of Nature. London: Taylor & Francis.

[B58] Jones C. Scholes L. Johnson D. Katsikitis M. Carras M. C. (2014). Gaming well: links between videogames and flourishing mental health. Front. Psychol. 5:260. 10.3389/fpsyg.2014.00260 24744743 PMC3978245

[B59] Kabat-Zinn J. (2003). Mindfulness-based interventions in context: past, present, and future. Clin. Psychol. Sci. Pract. 10, 144–156. 10.1093/clipsy.bpg016

[B60] Kennedy S. D. (2014). Technowellness: a new wellness construct in the 21st century. J. Couns. Leadersh. Advocacy 1, 113–127. 10.1080/2326716X.2014.902759

[B61] Kitson A. Riecke B. E. Vidyarthi J. (2014). Sonic cradle: investigating meditative aspects of an interactive technology, in NCE-GRAND 2014 Conference (Ottawa, ON), 1–4.

[B62] Kitson A. Schiphorst T. Riecke B. E. (2018). Are you dreaming?: a phenomenological study on understanding lucid dreams as a tool for introspection in virtual reality, in Proceedings of the 2018 CHI Conference on Human Factors in Computing Systems CHI'18 (New York, NY: ACM), 343:1–343:12.

[B63] Knowles B. (2013). Re-imagining Persuasion: designing for self-transcendence, in CHI'13 Extended Abstracts on Human Factors in Computing Systems CHI EA'13 (New York, NY: ACM), 2713–2718.

[B64] Kosunen I. Salminen M. Järvelä S. Ruonala A. Ravaja N. Jacucci G. (2016). RelaWorld: neuroadaptive and immersive virtual reality meditation system, in Proceedings of the 21st International Conference on Intelligent User Interfaces IUI'16 (New York, NY: ACM), 208–217.

[B65] Lee W. Lim Y. Shusterman R. (2014). Practicing somaesthetics: exploring its impact on interactive product design ideation, in Proceedings of the 2014 Conference on Designing Interactive Systems DIS '14 (Vancvouer, BC: ACM), 1055–1064.

[B66] Levac D. Colquhoun H. O'Brien K. K. (2010). Scoping studies: advancing the methodology. Implement. Sci. 5:69. 10.1186/1748-5908-5-69 20854677 PMC2954944

[B67] Macaranas A. Antle A. N. Riecke B. E. (2015). What is intuitive interaction? Balancing users' performance and satisfaction with natural user interfaces. Interact. Comput. 27, 357–370. 10.1093/iwc/iwv003

[B68] Maciosek M. V. Coffield A. B. Flottemesch T. J. Edwards N. M. Solberg L. I. (2010). Greater use of preventive services in U.S. health care could save lives at little or no cost. Health Aff. 29, 1656–1660. 10.1377/hlthaff.2008.0701 20820022

[B69] Markman A. B. Brendl C. M. (2005). Constraining theories of embodied cognition. Psychol. Sci. 16, 6–10. 10.1111/j.0956-7976.2005.00772.x 15660844

[B70] Mokdad A. H. Marks J. S. Stroup D. F. Gerberding J. L. (2004). Actual causes of death in the United States, 2000. JAMA 291, 1238–1245. 10.1001/jama.291.10.1238 15010446

[B71] Mossbridge J. (2016). Designing transcendence technology, in Psychology's New Design Science and the Reflective Practitioner, eds Imholz S. Sachter J. (River Bend, NC: LibraLab Press), 1–27.

[B72] Muñoz J. E. Paulino T. Vasanth H. Baras K. (2016). PhysioVR: A novel mobile virtual reality framework for physiological computing, in 2016 IEEE 18th International Conference on e-Health Networking, Applications and Services (Munich: Healthcom), 1–6.

[B73] Myers J. E. Sweeney T. J. (2005). The indivisible self: an evidence-based model of weliness. J. Individ. Psychol. 61, 269–279.

[B74] Navarro-Haro M. V. López-del-Hoyo Y. Campos D. Linehan M. M. Hoffman H. G. García-Palacios A.. (2017). Meditation experts try Virtual Reality Mindfulness: a pilot study evaluation of the feasibility and acceptability of virtual reality to facilitate mindfulness practice in people attending a Mindfulness conference. PLoS ONE 12:e0187777. 10.1371/journal.pone.0187777 29166665 PMC5699841

[B75] Nell V. (1988). Lost in a Book: The Psychology of Reading for Pleasure. New Haven, CT: Yale University Press.

[B76] Norman D. A. (2004). Emotional Design: Why We Love (or Hate) Everyday Things. New York, NY: BasicBooks.

[B77] Norman D. A. Draper S. W. (1986). User Centered System Design; New Perspectives on Human-Computer Interaction. Hillsdale, NJ: L. Erlbaum Associates Inc.

[B78] Pasch M. Bianchi-Berthouze N. van Dijk B. Nijholt A. (2009). Immersion in movement-based interaction, in Intelligent Technologies for Interactive Entertainment Lecture Notes of the Institute for Computer Sciences, Social Informatics and Telecommunications Engineering, eds Nijholt A. Reidsma D. Hondorp H. (Berlin; Heidelberg: Springer), 169–180.

[B79] Patibanda R. Mueller F. “Floyd,” Leskovsek M. Duckworth J. (2017). Life tree: understanding the design of breathing exercise games, in Proceedings of the Annual Symposium on Computer-Human Interaction in Play CHI PLAY'17 (New York, NY: ACM), 19–31.

[B80] Picard R. W. (1995). Affective computing. Cambridge, MA: MIT Press.

[B81] Picard R. W. (2010). Emotion research by the people, for the people. Emot. Rev. 2, 250–254. 10.1177/1754073910364256

[B82] Plaza I. Demarzo M. M. Herrera-Mercadal P. García-Campayo J. (2013). Mindfulness-based mobile applications: literature review and analysis of current features. JMIR MHealth UHealth 1:e24. 10.2196/mhealth.2733 25099314 PMC4114453

[B83] Prpa M. Cochrane K. Riecke B. E. (2015). Hacking alternatives in 21st century: designing a bio-responsive virtual environment for stress reduction, in Pervasive Computing Paradigms for Mental Health Communications in Computer and Information Science, eds Serino S. Matic A. Giakoumis D. Lopez G. Cipersso P. (Cham: Springer), 34–39.

[B84] Prpa M. Quesnel D. Vidyarthi J. Kitson A. Riecke B. E. (2016). Sonic Cradle — Immersive interaction design combining breathing– and neurofeedback to foster focused attention meditation on breath, in Poster Presented at the 2nd International Conference on Mindfulness (Rome).

[B85] Prpa M. Tatar K. Riecke B. E. Pasquier P. (2017). The pulse breath water system: exploring breathing as an embodied interaction for enhancing the affective potential of virtual reality, in Virtual, Augmented and Mixed Reality Lecture Notes in Computer Science, ed Shumaker R. (Cham: Springer), 153–172.

[B86] Quaglia J. T. Holecek A. (2018). Lucid virtual dreaming: antecedents and consequents of virtual lucidity during virtual threat, in Proceedings of IEEE Virtual Reality IEEEVR '18 (Reutlingen: IEEE).

[B87] Quesnel D. Riecke B. E. (2017). Awestruck: natural interaction with virtual reality on eliciting awe, in 2017 IEEE Symposium on 3D User Interfaces (3DUI) (Los Angeles, CA), 205–206.

[B88] Ramirez R. Vamvakousis Z. (2012). Detecting emotion from EEG signals using the emotive Epoc device, in Brain Informatics Lecture Notes in Computer Science, eds Zanzotto F. Tsumoto S. Taatgen N. Yao Y. Y. (Berlin; Heidelberg: Springer), 175–184.

[B89] Rettie R. (2004). Using Goffman's frameworks to explain presence and reality, in 7th Annual International Workshop on Presence (Valencia), 117–124.

[B90] Riva G. Baños R. M. Botella C. Mantovani F. Gaggioli A. (2016). Transforming experience: the potential of augmented reality and virtual reality for enhancing personal and clinical change. Front. Psychiatry 7:164. 10.3389/fpsyt.2016.00164 27746747 PMC5043228

[B91] Riva G. Baños R. M. Botella C. Wiederhold B. K. Gaggioli A. (2012). Positive technology: using interactive technologies to promote positive functioning. Cyberpsychology Behav. Soc. Netw. 15, 69–77. 10.1089/cyber.2011.0139 22149077

[B92] Riva G. Mantovani F. Capideville C. S. Preziosa A. Morganti F. Villani D.. (2007). Affective interactions using virtual reality: the link between presence and emotions. Cyberpsychol. Behav. 10, 45–56. 10.1089/cpb.2006.9993 17305448

[B93] Roo J. S. Gervais R. Hachet M. (2016). Inner garden: an augmented sandbox designed for self-reflection, in Proceedings of the TEI'16: Tenth International Conference on Tangible, Embedded, and Embodied Interaction TEI'16 (New York, NY: ACM), 570–576.

[B94] Rubio-Tamayo J. Gertrudix Barrio M. García García F. (2017). Immersive environments and virtual reality: systematic review and advances in communication, interaction and simulation. Multimodal Technol. Interact. 1:21 10.3390/mti1040021

[B95] Ryan R. M. Deci E. L. (2000). Self-determination theory and the facilitation of intrinsic motivation, social development, and well-being. Am. Psychol. 55, 68–78. 10.1037/0003-066X.55.1.68 11392867

[B96] Ryff C. D. (1989). Happiness is everything, or is it? Explorations on the meaning of psychological well-being. J. Pers. Soc. Psychol. 57, 1069–1081. 10.1037/0022-3514.57.6.1069

[B97] Sakamoto M. Nakajima T. Alexandrova T. (2015). Enhancing values through virtuality for intelligent artifacts that influence human attitude and behavior. Multimed. Tools Appl. 74, 11537–11568. 10.1007/s11042-014-2250-5

[B98] Sander T. (2011). Positive computing, in Positive Psychology as Social Change, ed Biswas-Diener R. (Dordrecht: Springer), 309–326.

[B99] Schiphorst T. (2009). Soft(N): toward a somaesthetics of touch, in CHI'09 Extended Abstracts on Human Factors in Computing Systems CHI EA'09 (New York, NY: ACM), 2427–2438.

[B100] Schultz P. W. Tabanico J. (2007). Self, identity, and the natural environment: exploring implicit connections with nature1. J. Appl. Soc. Psychol. 37, 1219–1247. 10.1111/j.1559-1816.2007.00210.x

[B101] Schwartz M. S. Andrasik F. (2017). Biofeedback: A Practitioner's Guide, 4th Edn New York, NY: Guilford Publications.

[B102] Seaborn K. A. (2016). Mixed Reality Gaming for Older Powered Chair Users: A Human Factors Model of Well-being and Engagement. Available online at: http://www.gregorydolinar.com/access.pdf

[B103] Seligman M. E. P. (2002). Authentic Happiness: Using the New Positive Psychology to Realize Your Potential for Lasting Fulfillment. New York, NY: Free Press.

[B104] Seligman M. E. P. (2012). Flourish : A Visionary New Understanding of Happiness and Well-being, 1st Edn New York, NY: Free Press.

[B105] Seligman M. E. P. Csikszentmihalyi M. (2014). Positive psychology: an introduction, in Flow and the Foundations of Positive Psychology, ed Csikszentmihalyi M. (Dordrecht: Springer), 279–298.

[B106] Shaw C. D. Gromala D. Seay A. F. (2007). The meditation chamber: enacting autonomic senses, in Proceedings of ENACTIVE/07 (Grenoble).

[B107] Shusterman R. (2012). Thinking Through the Body: Essays in Somaesthetics. New York, NY; Cambridge, UK Cambridge University Press.

[B108] Slater M. Wilbur S. (1997). A framework for immersive virtual environments (five): speculations on the role of presence in virtual environments. Presence Teleoperators Virtual Environ. 6, 603–616. 10.1162/pres.1997.6.6.603

[B109] Sliwinski J. Katsikitis M. Jones C. M. (2017). A review of interactive technologies as support tools for the cultivation of mindfulness, in Mindfulness (Springer), 1–10.

[B110] Spanakis E. G. Santana S. Tsiknakis M. Marias K. Sakkalis V. Teixeira A.. (2016). Technology-based innovations to foster personalized healthy lifestyles and well-being: a targeted review. J. Med. Internet Res. 18:e128. 10.2196/jmir.4863 27342137 PMC4938884

[B111] Steuer J. (1992). Defining virtual reality: dimensions determining telepresence. J. Commun. 42, 73–93. 10.1111/j.1460-2466.1992.tb00812.x

[B112] Triberti S. Riva G. (2016). Being present in action: a theoretical model about the “interlocking” between intentions and environmental affordances. Front. Psychol. 6:2052. 10.3389/fpsyg.2015.02052 26834670 PMC4722118

[B113] Triberti S. Chirico A. La Rocca G. Riva G. (2017). Developing emotional design: emotions as cognitive processes and their role in the design of interactive technologies. Front. Psychol. 8:1773. 10.3389/fpsyg.2017.01773 29062300 PMC5640767

[B114] Valmaggia L. R. Latif L. Kempton M. J. Rus-Calafell M. (2016). Virtual reality in the psychological treatment for mental health problems: an systematic review of recent evidence. Psychiatry Res. 236, 189–195. 10.1016/j.psychres.2016.01.015 26795129

[B115] van den Hoogen W. IJsselsteijn W. A. De Kort Y. A. (2009). Effects of Sensory Immersion on Behavioural Indicators of Player Experience: Movement Synchrony and Controller Pressure, in Conference DiGRA (West London, UK).

[B116] van Rooij M. Lobel A. Harris O. Smit N. Granic I. (2016). DEEP: a biofeedback virtual reality game for children at-risk for anxiety, in Proceedings of the 2016 CHI Conference Extended Abstracts on Human Factors in Computing Systems CHI EA'16 (New York, NY: ACM), 1989–1997.

[B117] Varela F. J. Rosch E. Thompson E. (1992). The Embodied Mind: Cognitive Science and Human Experience. Cambridge, MA: MIT Press.

[B118] Venter H. J. (2017). Self-transcendence: Maslow's answer to cultural closeness. J. Innov. Manag. 4, 3–7. Available online at: https://journals.fe.up.pt/index.php/IJMAI/article/view/367

[B119] Vidyarthi J. (2012). Sonic Cradle: Evoking Mindfulness Through Immersive Interaction Design. Available online at: https://vimeo.com/55230632

[B120] Vidyarthi J. Riecke B. E. (2014). Interactively mediating experiences of mindfulness meditation. Int. J. Hum. Comput. Stud., 72, 674–688. 10.1016/j.ijhcs.2014.01.006

[B121] Weiser M. Brown J. S. (1996). Designing calm technology. PowerGrid J. 1, 75–85.

[B122] Wiethoff A. Butz A. (2010). Colour vision: controlling light patterns through postures, in Proceedings of the 10th International Conference on Smart Graphics SG'10 (Berlin; Heidelberg: Springer-Verlag), 281–284.

