# Supplementary material for: Immersive Interactive Technologies for Positive Change: A Scoping Review and Design Considerations
Source: Front Psychol. 2018 Aug 3;9:1354. doi: 10.3389/fpsyg.2018.01354 (PMC6085587; doi:10.3389/fpsyg.2018.01354)
Supplement: Supplementary file 1 [file Table_1.PDF]

Type of XR and Technologies/Platforms Used

Soundscape		installation		Augmented Reality		Augmented Virtuality		Virtual Reality	
technology	platform	technology	platform	technology	platform	technology	platform	technology	platform
The Sensorium – Hinterberger (2011)		ColourVision – Wiethoff & Butz (2010)		Multisensory Interactive Window – Angelini et al. (2015)		Powered to Play – Seaborn (2016)		PsychicVR – Amores et al. (2016)	
surround speakers, EEG, projector, GSR, pulse, respiration	Thought Translation Device, PC, DMX control unit	camera, RGB lights	C++ video analysis software, DMX converter	touch screen, camera, diffuser, air blower	PQLab G4S multitouch overlay, HD camera, All-In-One Touchscreen PC, Google Chrome web & .NET app	smartphone	Android or iOS, Javascript framework (Appcelerator Titanium), Google Maps API	HMD, EEG	Oculus Rift, Interaxon Muse
Sonic Cradle (mod) – Kitson et al. (2014)		ORGONA – Bal (2013)		Catching Fireflies – Eubanks (2011)				Emotional Beasts – Bernal & Maes (2017)	
surround speakers, respiration	Thought Technology ProComp2, Max MSP	motion sensor, microphone, projector	Kinect, Arduino, Processing	smartphone	Android			HMD, PPG, GSR, mocap suit	HTC Vive, Shimmer3, Perception Neuron, Unreal
SOLAR – Prpa et al. (2015)		ORGONA Underwater – Bal (2013)		Inner Garden – Roo et al. (2017)				Cemetary & Park VE – Chittaro et al. (2017)	
surround speakers, respiration, EEG	Thought Technology ProComp2, Max MSP, Emotiv EPOC, Unity	motion sensor, microphone, projector	Kinect, Arduino, Processing	projector, respiration, HR, (HMD)	vvvv, Unity, Open Vibe, ASUS short-throw projector, Kinect v1, Mio Fuse smartwatch (Oculus Rift DK2)			HMD, PPG	SonyHMZ-T1, Thought Technology ProComp Infiniti
Sonic Cradle + – Prpa et al. (2016)		ORGONA Chakra – Bal (2013)		MikuMikuDance – Sakamoto et al. (2015)				Angkor Wat – Choo & May (2014)	
surround speakers, respiration, EEG	Thought Technology ProComp2, Max MSP, Interaxon Muse, Unity	motion sensor, projector	Kinect, Processing	projectors, cameras, motion sensor	Kinect, OpenNI, Skype			HMD, EEG	Oculus Rift DK2, Emotiv EPOC
Sonic Cradle – Vidyarthi (2012)		ORGONA Prana – Bal (2013)		Virtual Aquarium – Sakamoto et al. (2015)				Osmose – Davies & Harrison (1996)	
surround speakers, respiration	Thought Technology ProComp2, Max MSP	motion sensor, projector	Kinect, Processing	accelerometer, mirror display	3-axis accelerometer			HMD, respiration vest, posture sensors	Division DVisor HMD, Polhemus Fastrak, custom breathing vest
								Strata – Du Plessis (2017)	
								HMD, EEG ,GSR, HR, respiration	Oculus CV1, Interaxon Muse, custom conductive band
								Realisitic Avatar Communication – Garau et al. (2003)	
								CAVE, HMD	ReaCT by Trimention, Silicon Graphics Onyx w/ two Polymus Fastraks, DIVE software
								Virtual Sophrologist – Gu & Frasson (2017)	
								HMD, EEG	EMOTIV EPOC EEG, Windows Unity Server UDP to VR, Samsung Gear VR
								Virtual Meditative Walk – Gromala et al. (2015)	
								Stereoscopic viewer, GSR	DeepStream, GSR fingerclip
								RelaWorld – Kosunen et al. (2016)	
								HMD, EEG	Oculus Rift DK2, QuickAmp, Open Vibe framework
								EmoCat – Muñoz et al. (2016)	
								HMD, smart watch, headphones, gamepad	LG G Watch R, Samsung Galaxy S4 w/low-cost HMD, PhysioVR, Unity
								VR DBT – Navarro-Haro et al. (2017)	
								HMD	Oculus Rift DK2
								Life Tree – Patibanda (2017)	
								HMD, microphone	Smartphone w/ HMD, respiration headset
								Pulse Breath Water – Prpa et al. (2017)	
								HMD, respiration belt	Oculus Rift DK2, Thought Technology, Max MSP patch, M+M middleware, Unity
								Google Earth VR – Quesnel & Riecke (2017)	
								HMD, camera	HTC Vive, Logitech HD C270 webcam and LEDs casting unidirectional light
								DEEP – van Rooij (2016)	
								HMD, respiration	Oculus Rift DK1/2
								Meditation Chamber – Shaw et al. (2007)	
								HMD, GSR, respiration, pulse	VFX-3D (interactive imaging systems), Thought Technology ProComp+, SVE Toolkit

1. source and full reference	2. description and name of the immersive interactive system	3. relevance to well-being and positive functioning	4. type of XR	5. technology used	6. platform	7. target user	8. number of users in study	9. input / output modalities	10. design elements and interaction strategies used	11. outcome	12. how design elements and interaction strategies contributed to support positive change and/or elicit positive states
Angelini, L., Caon, M., Couture, N., Khaled, O. A., & Mugellini, E. (2015). The Multisensory Interactive Window: Immersive Experiences for the Elderly. In Adjunct Proceedings of the 2015 ACM International Joint Conference on Pervasive and Ubiquitous Computing and Proceedings of the 2015 ACM International Symposium on Wearable Computers (pp. 963–968). New York, NY, USA: ACM. https://doi.org/10.1145/2800835.2806209	Multisensory Interactive Window	enhance the older adult's well-being at home	AR, tangilbes	touch screen, camera	PQLab G4S multitouch overlay, HD camera, All-In-One Touchscreen PC, Google Chrome web app, .NET app	older adults	N/A	input: opening physical window, touch gestures, weather API; output: telepresence audio/visuals, open/close virtual binds, smell and air blower.	connection, nature elements, social presence	Proof of concep: human-to-human connection, increased well-being	Connecting with others remotely and discovering different places in the world.
Amores, J., Benavides, X., & Maes, P. (2016). PsychicVR: Increasing Mindfulness by Using Virtual Reality and Brain Computer Interfaces. Proceedings of the 2016 CHI Conference Extended Abstracts on Human Factors in Computing Systems, CHI EA '16 (pp. 2–2). New York, NY, USA: ACM. doi:10.1145 /2851581.2889442	PsychicVR: virtually create and control fire through concentration	improve mindfulness	VR, biofeedback	HMD, EEG	Oculus Rift, Muse EEG	humans	N/A	input: brain electrical activity; output: virtual fire created and levitates up with higher concentration	concentrate more = visual feedback; superhero metaphor	Proof of concept	Increase mindfulness through concentrating on making a virtual object levitate. Playful design through immersive environment and having superpowers
Bal, H. (2013). Responsive Aesthetics for Yogic Meditation: An Innovative Design Theory for Holistic Health that supports Autonomy and Effective Training (M.Des.). Canada: Ontario College of Art & Design (Canada). Retrieved from https://search-proquest-com.proxy.lib.sfu. ca/docview/1490793959/abstract/92434D3FE69845F4PQ/3	ORGONA: using your breath, you can virtually propel a cloth into space	breathing practices, borrowed from meditation and yoga practices, to foster physical and psychological empowerment	projection, biofeedback	motion sensor, microphone, projector	Kinect, Arduino, Processing	individuals who face mental and physical disorders	N/A	input: exhaling and x-axis bodily motion; output: cloth floats higher and background scrolls up when exhaling, and down when no input. Move to get under the cloth.	breath awareness and embodied physical empowerment	Proof of concept	Mind-body dialogues: a calm body breeds a calm mind. The gamified aspect allows a state of flow to create conditions for increased well-being.
	ORGONA Underwater: using your breath, create ripples in a virtual underwater	exercise of healthy behaviour	projection, biofeedback	motion sensor, microphone, projector	Kinect, Arduino, Processing	individuals who face mental and physical disorders	N/A	input: exhaling and x-axis bodily motion; output: cloth floats higher and background scrolls up when exhaling, and down when no input. Move to get under the cloth.	breath awareness and embodied physical empowerment	Prototype	Mind-body dialogues: a calm body breeds a calm mind. The gamified aspect allows a state of flow to create conditions for increased well-being.
	Interactive Chakra-based Breathwork: visualize your breath as a virtual outline of a person with an orb of energy	focus on breathing and relaxation from meditation practices	projection in dark room, biofeedback	motion sensor, projector	Kinect, Processing	individuals who face mental and physical disorders	5	input: inhaling with arms up --> inhale sound, outline of virtual body mirrored, fireflies move inward, and circle decreases into the virtual body. exhaling with arms down --> exhale sound, outline of virtual body lights up with chakras, fireflies move outward, and circle increases outward	visual and auditory cues help user breath smoothly and deeply. gradually, the exhales move through the chakras until only the breath is left – abstract orb that responds to breathing, representing transcendence.	Strong calming effect; Reprogramming of clarity; Awareness to breathing; Visuals and audio helpful indictors of progress; Arm movements natural way to signify breathing; Circle visuals should be reversed to be congruent with actual body expanding/contracting during breathing; Music should be simple and realted to holistic health.	Cathartic healing through breathwork and gradual transcendence provided by natural bodily movements and visual/auditory feedback to show performance.
	ORGONA Prana: interation of above system with extended narrative and more auditory feedback	focus on breathing and relaxation from meditation practices	projection in dark room, biofeedback	motion sensor, projector	Kinect, Processing	individuals who face mental and physical disorders	5	same as above expect with cosmos background imagery moving clockwise and counter-clockwise with inhalation and exhalation, respectfully. Chakras disappear first, then reappear in reverse order to complete the experience.	Same as above; repeated in reverse order of chakras.	Positive experience; relaxing; visuals good feedback for performance; audio can be intrusive; End of experience unclear.	Use of visual feedback that was both abstract and related to spiritual practices of bodily sensations and chakras helped users to relax and focus on their breath.
Bernal, G., & Maes, P. (2017). Emotional Beasts: Visually Expressing Emotions Through Avatars in VR. Proceedings of the 2017 CHI Conference Extended Abstracts on Human Factors in Computing Systems, CHI EA '17 (pp. 2395–2402). New York, NY, USA: ACM. doi:10.1145/3027063.3053207	Emotional Beasts: affective expression in social VR	fostering deep, emotionally compelling human-to-human connection	VR, biofeedback	HMD, PPG, GSR, mocap sensor suit	HTC Vive VR headset, Shimmer3 sensor, Perception Neuron suit, Unreal Engine	humans	N/A	input: heart rate, skin conductance (emotional arousal); output: reactive fur and particle system brightness/colour	Visually express emotions. VR avatars project non-verbal cues form the wearer's emotional state	Proof of concept	VR avatars project non-verbal cues from the wearer's emotional state, so that virtual users can both communicate and perceive emotions in social VR
Chittaro, L., Sioni, R., Crescentini, C., & Fabbro, F. (2017). Mortality salience in virtual reality experiences and its effects on users' attitudes towards risk. International Journal of Human-Computer Studies, 101, 10–22. doi:10.1016/j.ijhcs.2017.01.002	Cemetary and Park VE: using VR to elicit morality salience	morality salience is linked to increasing positive health and well-being, including mindfulness	VR, biofeedback	HMD, PPG	SonyHMZ-T1 stereoscopic HMD, Thought Technology ProComp Infiniti encoder	humans	108 (95 Female)	input: Nintendo Nunchuck joystick; output: movement in VE	Visual morality cues such as tombs, burialrecesses, and a group of mourners attending a funeral	Cemetary VE elicited death related themes compared to park VE; increased risk perception for both the self and others; Harm Avoidance as a personality factor is a mediating trait for risk perception; mindfuless personality not a factor; heart rate variability was increased.	VR can elicit morality salience through visual cues and immersive properties
Choo, A., & May, A. (2014). Virtual Mindfulness Meditation Virtual Reality and Electroencephalography for Health Gamification. 2014 Ieee Games, Media, Entertainment (gem).	Angkor Wat: virtual guided seated meditation that is gamified for learning mindfulness	mindfulness has many health benefits, so training can help encourage positive functioning	VR, biofeedback	EEG	Emotiv EPOC EEG	all who would like to learn mindfulness meditation	N/A	input: brain electrical activity; output: virtual flowers bloom in VE	The more flowers in bloom, the higher the player's meditative scores. A lower meditation score will have fewer flowers in bloom at any given time	Proof of concept	Use of visual feedback shows learning progress of meditation, which may accelerate the postive benefits of meditation. Calming visuals and audio contribute to relaxed state.
Davies, C., & Harrison, J. (1996). Osmose: Towards Broadening the Aesthetics of Virtual Reality. SIGGRAPH Comput. Graph., 30 (4), 25–28. doi:10.1145/240806.240808	Osmose: a virtual environment that allows users to shed their habitual ways of looking at(and behaving in) the world.	self-transcendence: the goal is to experience the real world in a fresh way, reawakening a fundamentalsense of their own "being-in-the-world."	VR, biofeedback	HMD, Respiration vest	Division DVisor HMD, Polhemus Fastrak, custom breathing vest, Onyx RealityEngine2, softimage SAAPHIRE and DKit development libraries, SGI's Performer and GL graphics libraries	humans	N/A	input: breath, posture tilt; output: movement vertical, movement horizontal	Emphasis on "being" rather than "doing. Attain full-body immersion through meditation and tai chi practices. Creating a sense of floating through breath and balance. Breath in to move up, breath out to move down; tilt body in direction you want to move horizontally. Evocative audio and human voices to reaffirm the role of the physical body inthe virtual space.	Feeling calm, a loosening of the boundariesbetween self and world, simultaneous feeling of disembodied (because of the visual aesthetic, being able to float and pass throughthings) and embodied (due to reliance on breath and balance, emotional, euphoric.	By enabling people to experience the unusual sensations ofseeing and floating through things, they are freed of their usual, habitual ways ofbeing in the world: this effect, in combination with the use of breath, solitaryimmersion and metaphorical content, appears to induce heightened awareness.
Du Plessis, I. (2017). Strata: A Biometric VR Experience. ACM SIGGRAPH 2017 VR Village, SIGGRAPH '17 (p. 14:1–14:2). New York, NY, USA: ACM. doi:10.1145/3089269.3089273	Strata: a biometric VR experience	immersive experience connects us to our own emotional state, teaching us to calm and focus our minds.	VR, biofeedback	HMD, EEG, GSR, HR, breathing	Oculus CV1, Muse EEG, custom conductive band	humans	N/A	input: brain electrical activity, heart rate, skin conductance, breathing rate; output: levitate upward through five fantastical worlds as you reach meditative state, biometrics change audio and visual components.	Mindfulness training fo rstress alleviation, meditation, anger management, empathy counseling.	Proof of concept	Reach meditative state and levitate upward --> reaching higher state of consciousness, letting go of distractive thoughts. See visuals and audio change to gain introspection on own internal states.
Eubanks, A. (2011). Catching Fireflies: A Persuasive Augmented Reality Game for Android Phones. (V. A. Clincy, Ed.)Proceedings of the 49th Annual Association for Computing Machinery Southeast Conference (acmse '11), 363–364.	Catching Fireflies: an AR game designed to draw people outdoors and appreciate nature.	Calming, nostolgic experience	AR	smartphone	Android	humans	N/A	input: camera, GPS coordinates, touch screen; output: vibration, animation of capturing firefly	Besides the camera and GPS, the application uses sound, vibration, the proximity sensor, accelerometers, and orientation sensors to encourage players to relive their outdoor childhood memories of catching fireflies.	Nostolgia, getting outdoors, appreciation of nature, movement	The app encourages players to go outside and partake in an activity they might not be able to do otherwise – catch fireflies. It is a playful way to get people to interact with the real environment and move their bodies, which has positive physical and psychological effects.
Garau, M., Slater, M., Vinayagamoorthy, V., Brogni, A., Steed, A., & Sasse, M. A. (2003). The Impact of Avatar Realism and Eye Gaze Control on Perceived Quality of Communication in a Shared Immersive Virtual Environment. Proceedings of the SIGCHI Conference on Human Factors in Computing Systems, CHI '03 (pp. 529–536). New York, NY, USA: ACM. doi:10.1145/642611. 642703	Communicating with realistic avatars in immersive virtual environments	Increasing social presence and communication	VR	CAVE, HMD	ReaCTor by Trimention, Silicon Graphics Onyx with two Polhemus Fastraks, DIVE software	humans	48 (24 Female)	input: analogue joystick, 5 button 3D mouse, head tracking device; output: movement, head position and orientation	Use of high-realism avatars and non-random gaze will increase social presence and communication in VEs	Inferred gaze high-realism avatar helps increase social presence and ease of communication	More realistic avatars and gazing at us can help increase social presence, in turn having a positive impact on communication in social VR settings.
Gromala, D., Tong, X., Choo, A., Karamnejad, M., & Shaw, C. D. (2015). The Virtual Meditative Walk: Virtual Reality Therapy for Chronic Pain Management. Proceedings of the 33rd Annual ACM Conference on Human Factors in Computing Systems, CHI '15 (pp. 521–524). New York, NY, USA: ACM. doi:10.1145/2702123. 2702344	Virtual Meditative Walk: enable chronic pain patients to learn Mindfulness-based stress reduction (MBSR)	Reduce pain and increase psychological health	VR, biofeedback	Stereoscopic viewer, GSR	DeepStream VR, GSR finger clips	chronic pain patients	6 (3 Female)	input: GSR arousal levels; output: virtual weather system	light fog in the forest, for example, recedes as a patient's GSR levels start to stabilize in favor of a mindful state. Alternatively, the fog thickens and draws closer when the patient's arousal levels increase.	pain level decreased after the VR MBSR experience compared to the control	Biofeedback helped users more easily learn mindfulness practices and provide a distraction from pain, which in turn decreased their overall pain over time.
Gu, G., & Frasson, C. (2017). Virtual Sophrologist: A Virtual Reality Neurofeedback Relaxation Training System. In Brain Function Assessment in Learning (pp. 176–185). Springer, Cham. https: //doi.org/10.1007/978-3-319-67615-9_16	Virtual Sophrologist: VR and EEG relaxation training system	Increased relaxation	VR, biofeedback	HMD, EEG	EMOTIV EPOC EEG, Windows Unity Server UDP to VR, Samsung Gear VR	humans	6 (2 Female)	input: brain waves; output: virtual text and audio feedback	physiological measures, mindfulness-meditation, nature elements	relaxed, increased mindfulness, decreased stress/anxiety	VEs support a calming environment: Seaside, Japanese Garden and Waterfall. Relaxation feedback keeps participants in the present moment.
Hartnett, J., Lin, P., Ortiz, L., & Tabas, L. (2006). A Responsive and Persuasive Audio Device to Stimulate Exercise and Fitness in Children. CHI '06 Extended Abstracts on Human Factors in Computing Systems, CHI EA '06 (pp. 1837–1842). New York, NY, USA: ACM. doi:10.1145/1125451.1125799	Immersive soundscape where users control and vary music tempo based on measured activity level.	Help stimulate exercise and fitness	immersive soundscape	Audio device, pedometer	TBD	children aged 8-11	15	input: movement; output: music	Increase movement --> increase music tempo; decrease movement --> decrease music tempo	Positive reception from concept testing	Moving the body to music (dancing) is a fun activity that also promotes both physical and mental health. Children are active without imposing stricter educational means of activity.
Hinterberger, T. (2011). The Sensorium: A Multimodal Neurofeedback Environment. Adv. in Hum.-Comp. Int., 2011, 3:1–3:10. doi:10.1155/2011/724204	Sensorium: a neurofeedback environment that allows people to experience signals from their nonperceptible body processes visually and auditorily	Improving bodily awareness and self-regulation to promote positive change	immersive soundscape and light room, biofeedback	Projector, surround speakers, EEG, skin conductance, pulse finger clip, repirator sensor	Thought Translation Device, PC, DMX cnotrol unit	humans	20 (11 Female)	input: brain electrical activity, heart rate, skin conductance, breathing; output: changes in light and sound	Positive slow potential shifts as generated on the cortex in relaxing moments should lead to a blue coloured environment while negative shifts normally reflect attentive moments in which the brain produces a readiness state (i.e., the "Bereitschaftspotenzial") which turned the environment into red.	Almost all of them reported an increase in contentment, relaxation, happiness, and inner harmony. They also reported a widening in their body consciousness.	Use of colours corresponding to certain emotional states (blue/red). Visual and auditory representation of internal states provide concrete feedback, so users can change their states and see that automatically if successful or not.
Janssen, J. H., van den Broek, E. L., Westerink, J. H., D, & M. (2012). Tune in to your emotions: a robust personalized affective music player. User Modeling and User - Adapted Interaction; Dordrecht, 22(3), 255–279. doi:http://dx.doi.org.proxy.lib.sfu.ca/10. 1007/s11257-011-9107-7	Affective Music Player (AMP): selects music for mood enhancement based on biofeedback	Improve or enhance mood	immersive soundscape, biofeedback	Headphones	Philips SBC HP400 headphone, NeXus-10 apparatus of Mind Media b.v.	humans	3 Males	input: skin temperature; output: corresponding music of mood	decreasing skin temperature --> positive mood song; increasing skin temperature --> negative mood song	The validation of the AMP was successful. The songs selected to reduce skin temperature did indeed decrease skin temperature.	Personalized music model that was first trained through regression and kernel density estimation, and then used as a way to guide affect (mood).
Kitson, A., Riecke, B. E., & Vidyarthi, J. (2014). Sonic Cradle: Investigating Meditative Aspects of an Interactive Technology (pp. 1–4). Presented at the NCE-GRAND 2014 Conference, Ottawa, Canada. Retrieved from http://eprints.iat.sfu.ca/1346/	Sonic Cradle: an interactive system designed to encourage a meditative attentional pattern akin to mindfulness	Encourages people to self-regulate and manage their stress in a healthy way	immersive soundscape, biofeedback	respiration physiological sensor, surround-sound	Max MSP, Thought Technology ProComp2	novice meditators	30	Input = breathing from abdomen and chest; hold breath to add sounds, breath quickly to subtract sounds	Mindfulness practice --> Sonic Cradle Interaction<br>Calm focused attention --> focus on respiratory control through sound interaction<br>Wandering mind --> Wandering mind sounds change<br>Meta-awareness --> Autonomic breathing as attention is brought back to sounds and user wonders how their breathing controls it	Decentering: "awareness of one's experience with some distance and disidentification rather than being carried away by one's thoughts and feelings". The increased decentering score we found after Sonic Cradle shows that participants were able to emotionally detach themselves from their thoughts and feelings while still acknowledging that they are there.	Playful, non-invasive made experience inviting. Dark room with only soundscape decreased distractions and allowed focus on breathing and present moment. Selected sounds were meditative in nature – neutral or calm yet still energetic enough to keep attention. Easy to learn mapping to explore breath. Auditory feedback on performance.
Kosunen, I., Salminen, M., Järvelä, S., Ruonala, A., Ravaja, N., & Jacucci, G. (2016). RelaWorld: Neuroadaptive and Immersive Virtual Reality Meditation System. Proceedings of the 21st International Conference on Intelligent User Interfaces, IUI '16 (pp. 208–217). New York, NY, USA: ACM. doi:10.1145/2856767. 2856796	RelaWorld: a neuroadaptive virtual reality meditation system that combines virtual reality with neurofeedback to provide a tool that is easy for novices to use yet provides added value even for experienced meditators.	Meditation in general and mindfulness in particular have been shown to be useful techniques in the treatment of a plethora of ailments	VR, biofeedback	HMD, EEG	Oculus Rift DK2, QuickAmp, Open Vibe framework	novice meditators	43 (26 Female)	input: brain electrical activity; output: virtual levitation and opacity of energy bubble around user	Increase in the power of the theta band (concentration) will produce floating; increase in the alpha band (relaxation) will increase the opacity of the energy bubble surrounding the user.	RelaWorld system elicits deeper relaxation, feeling of presence and a deeper level of meditation when compared to a similar setup without head-mounted display or neurofeedback.	the head-mounted display generates higher meditative states when compared to the same system displayed on a normal screen. Neurofeed- back further enhances the experience, providing higher levels of presence as reported by the users.
Muñoz, J. E., Paulino, T., Vasanth, H., & Baras, K. (2016). PhysioVR: A novel mobile virtual reality framework for physiological computing. 2016 IEEE 18th International Conference on e-Health Networking, Applications and Services (Healthcom) (pp. 1–6). Presented at the 2016 IEEE 18th International Conference on e-Health Networking, Applications and Services (Healthcom). doi:10.1109/HealthCom.2016.7749512	EmoCat Rescue: encourages players to regularize their heartbeats in order to find a cat lost in a forest.	Encourages deep breathing in order to lower heart rate, ultimately increasing health	VR, biofeedback	HMD, smartwatch, headphones, gamepad	LG G Watch R, Samsung Galaxy S4 w/ low-cost HMD, PhysioVR, Unity	humans	N/A	input: heart rate, gamepad; output: movement, cat's meow	Lower your HR in order to hear a virtual cat meow, so that you can find it and rescue the cat. Deep breathing is encouraged to lower HR through gamification techniques	Proof of concept	Gamification and real-time visual and auditory feedback on internal states help people learn to regulate their internal states.

1. source and full reference	2. description and name of the immersive interactive system	3. relevance to well-being and positive functioning	4. type of XR	5. technology used	6. platform	7. target user	8. number of users in study	9. input / output modalities	10. design elements and interaction strategies used	11. outcome	12. how design elements and interaction strategies contributed to support positive change and/or elicit positive states
Navarro-Haro, M. V., López-del-Hoyo, Y., Campos, D., Linehan, M. M., Hoffman, H. G., García-Palacios, A., Modrego-Alarcón, M., et al. (2017). Meditation experts try Virtual Reality Mindfulness: A pilot study evaluation of the feasibility and acceptability of Virtual Reality to facilitate mindfulness practice in people attending a Mindfulness conference. PLoS One; San Francisco, 12(11), e0187777. doi:http://dx.doi.org.proxy.lib.sfu.ca/10.1371/journal.pone.0187777	VR Dialectical Behavioral Therapy (DBT®): mindfulness skills training technique	Regular mindfulness practice benefits people both mentally and physically	VR	HMD	Oculus Rift DK2	people who have trouble focusing attention	44 (28 Female)	input: head movement; output: virtual view	Nature visuals and sounds matched a guided meditation instruction, where the participant could follow along and practice focusing and paying attention with minimal distractions and concrete things to focus on.	participants reported significantly less sadness, anger, and anxi- ety, and reported being significantly more relaxed	VR is a highly controlled world that allows the user to focus on the essentials during meditation and blocks out other distractions. This is good for those who have trouble focusing their attention or more traditional forms of meditation do not work.
Patibanda, R., Mueller, F. "Floyd," Leskovsek, M., & Duckworth, J. (2017). Life Tree: Understanding the Design of Breathing Exercise Games. Proceedings of the Annual Symposium on Computer-Human Interaction in Play, CHI PLAY '17 (pp. 19–31). New York, NY, USA: ACM. doi:10.1145/3116595.3116621	Life Tree: a virtual reality (VR) game in which a player controls the growth of a tree by practicing pursed-lip breathing.	Regular breathing exercises can be a beneficial part of leading a healthy life.	VR, biofeedback	HMD, microphone	Smartphone + HMD, Breathing+ headset	humans	32 (16 Female)	input: pursed lip breathing; output: virtual tree growth	Inhaling expands tree, and exhaling contracts tree. Sitting (phone accelerometer) will trigger tree to be submerged in water. Leaves are blown on exhalation. Background rhythmic breathing to help players match their breathing.	Overall, participants liked playing Life Tree. They reported that "the game was very fascinating and de-cluttering". They also thought of it as a novel experience. P17, a female participant said, "It is really good. I feel surprised about that game because I have not experienced such a kind of game before. It really felt special for me".	Design considerations listed: 1. Consider Using Subtle Onboarding to Help Players Engage with the Breathing Exercises; 2. Consider Using Non-Interruptive Breathing Feedback to Support Self-Awareness of Breathing During Gameplay; 3. Consider Using the Objects in the Game Environment to Provide Imitative Breathing Feedback; 4. Consider Using a Minimalist Approach to Designing Naturalistic Visuals to Help Players Focus on their Breathing; 5. Consider the Intimate Placement of Breathing Hardware and how it can Affect Breathing Performance; 6. Consider Designing Breathing Hardware that Helps Players Hear their Own Breathing.
Prpa, M., Cochrane, K., & Riecke, B. E. (2015). Hacking Alternatives in 21st Century: Designing a Bio-Responsive Virtual Environment for Stress Reduction. Pervasive Computing Paradigms for Mental Health, Communications in Computer and Information Science (pp. 34–39). Presented at the International Symposium on Pervasive Computing Paradigms for Mental Health, Springer, Cham. doi:10.1007/978-3-319-32270-4_4	SOLAR: immersive virtual environment (VE) that assists novice users to learn the stress reducing practice of mindfulness meditation	Meditation practice is known to reduce stress	soundscape, biofeedback	EEG, respiration belt	Emotiv Epoc, Thought Technology ProComp2, Unity, Max MSP	novice meditators	13	input: brain electrical activity, respiration; output: meditation sounds and visuals	In SOLAR, the user is rewarded with a complex soundscape when they are taking deep breaths from their diaphragm. If the user begins to breath from their chest (above their thorax) or starts taking shallow breaths, the soundscape becomes simplified. In the visuals, the respiration sensors are mapped to the "breathing circle" (in front of the silhouette). The breath circle becomes larger and smaller as the user inhales and exhales.	Participants felt relaxed	The following design principles were applied: 1. thought distracting - distancing from negative thoughts; 2. abstract visual elements - pleasant visual feedback; 3. rewarding system - motivational feedback; 4. Immersive and Attention Restorative Environments - nature elements.
Prpa, M., Quesnel, D., Vidyarthi, J., Kitson, A., & Riecke, B. E. (2016). Sonic Cradle — Immersive inter-ac-tion design com-bin-ing breath-ing– and neu-ro-feed-back to foster focused atten-tion med-i-ta-tion on breath. Presented at the 2nd International Conference on Mindfulness, Rome, Italy.	Sonic Cradle +: a system that encourages deep diaphragm (abdominal) breathing by providing real-time neuro- and biofeedback generated from EEG and respiration data.	Mindfulness meditation practice is widely recognized for its psycho-logical and physical well-being benefits	soundscape, biofeedback	EEG, respiration belt, surround-sound	Interaxon Muse, Max MSP, Thought Technology ProComp2	novice meditators	8	input: brain activity, respiration; output: sound	When EEG data reveals a state of distraction, the soundscape becomes more salient, increasing its ability to cue users back to their breath with curiosity. Once they achieve focused attention, the interaction paradigm fades out, allowing users to meditate undisturbed.	Self-reported anxiety and arousal levels decreased, and pleasantness increased	Sounds and physical set-up was relaxing and had minimal distractions that allowed focus on meditation. Biofeedback gave users a sense of their performance and gently guided them back to their breath when distracted. The revisions could include instructions to emphasize reflection on 'calmness', which is suggestive of mindfulness practices' focus of inner sensations opposed to a quantification of performance.
Prpa, M., Tatar, K., Riecke, B. E., & Pasquier, P. (2017). The Pulse Breath Water System: Exploring Breathing as an Embodied Interaction for Enhancing the Affective Potential of Virtual Reality. Virtual, Augmented and Mixed Reality, Lecture Notes in Computer Science (pp. 153–172). Presented at the International Conference on Virtual, Augmented and Mixed Reality, Springer, Cham. doi: 10.1007/978-3-319-57987-0_13	Pulse Breath Water: immersive virtual environment (VE) with affect estimation in sound	the integrity of our minds and bodies engaged in actions with environments, in a process through which meaning and understanding are generated.	VR, biofeedback	HMD, respiration belt	Oculus Rift DK2, Thought Technology, Max MSP patch, M+M middleware, Unity	humans	24 (16 Female)	input: respiration rate, arousal; output: user position vertically, wave amplitude	Breathing frequency as well as eventfulness (arousal) and pleasantness (valence) levels of the audio environment are sent from Max msp patch to the game engine Unity 3D. In Unity, the value of eventfulness is mapped to the waves of the ocean. Higher aroused states result in a more disturbed ocean surface and waves. The colour of the sky progresses from grey (at the beginning of the experience) to pitch black (at the end of a session) over the span of eighth minutes. A participant's breathing data controls the elevation of the user in the VE in that, when the user breathes in, their position in the environment is elevated so they can rise above the ocean surface. Similarly, when the participant exhales, they sink.	Relaxed, calm, engaged, breath awareness	Familiarity First, Engagement After; Tension and Relaxation, at the Same Time; Environmental Context Is the Key
Quesnel, D., & Riecke, B. E. (2017). Awestruck: Natural interaction with virtual reality on eliciting awe (pp. 205–206). IEEE. doi: 10.1109/3DUI.2017.7893343	Google Earth VR: flying interface to explore the Earth, leading to the experience of awe	transformative experiences, the feeling of awe is found to alter an individual's perception in positive, lasting manners.	VR	HMD, camera	HTC Vive, Sennheiser noise-cancelling headphones, Logitech HD C270 webcam and LEDs casting unidirectional light	humans	16 (6 Female)	input: HTC Vive wands; output: flight in 3D	connection with the planet; movement by flying with natural interation (use of bean bag chair), nature elements – Earth from space and zooming into famous places or home town	43.8% of the participants experienced goose bumps; if they experienced goose bumps they were more likely to experience awe. Females were more likely to expereince goose bumps (but not awe) than males. No differences in flight vs. standing conditions.	interactive VR can induce both subjective and physiological indicators of awe. embodied interfaces must have low cognitive load and inarduous interactions to be successful.
Roo, J. S., Gervais, R., Frey, J., & Hachet, M. (2017). Inner Garden: Connecting Inner States to a Mixed Reality Sandbox for Mindfulness. Proceedings of the 2017 CHI Conference on Human Factors in Computing Systems, CHI '17 (pp. 1459–1470). New York, NY, USA: ACM. doi:10.1145/3025453.3025743	Inner Garden: augmented sandbox to support mindfulness practices	Inspire self-motivation and curiosity	AR, VR, biofeedback	HMD, projector, respiration belt, HR monitor	vvvv, Unity, OpenViBE, ASUS short-throw projector, Kinect v1, Mio Fuse smartwatch, Oculus Rift DK2	humans	12 Females	input: scuplting sand, breathing, HR; output: garden appearance	oscillating breathing patterns are mapped to the water level (which creates waves), and to the wind strength (creating gusts of wind); Cardiac coherance was used to indicate garden health. When in good health, the amount of clouds reduces, flora's growth speed is increased and sounds caused by the fauna are more present. Note that there is no obvious "unhealthy" state; fauna might hide, but trees will not start dying as a result of a low CC score.<br>The following design considerations were used: balance distraction and guidance, keep it minimalist, be non-judgmental and non-striving, promote acceptance and autonomy, use tagible interaction, choose the right reality.	They tested the system with meditation practitioners of different levels of experience (from initiated to daily meditators), which found the design engaging while also being well suited for mindfulness. Preliminary quantitative results seem to indicate that the system foster a calm and mindful state on the users.	Different mediums used for greater accessability: AR for tangible interaction and "trying it out"; VR for more immersive and personal experience and a chance to go deeper. Nature elements and biofeedback set the stage for a relaxing and inviting experience.
van Rooij, M., Lobel, A., Harris, O., Smit, N., & Granic, I. (2016). DEEP: A Biofeedback Virtual Reality Game for Children At-risk for Anxiety. Proceedings of the 2016 CHI Conference Extended Abstracts on Human Factors in Computing Systems, CHI EA '16 (pp. 1989–1997). New York, NY, USA: ACM. doi:10.1145/2851581.2892452	DEEP: a virtual reality (VR) game that situates players in a beautiful underwater fantasy world in which they can move around freely and explore at their leisure	provide an immersive and relaxing experience in order to decrease anxiety	VR, biofeedback	HMD, variable resistor/stretch sensor	Oculus DK	children at risk for anxiety	86 (42 Female)	input: respiration; output: expanding and contracting circle	Large circle --> inhilation peak, small circle --> exhilation peak; lung capacity <=50% --> gravity applied; inhaling --> upward movement, exhaling --> downward movement	the pilot study demonstrated that playing DEEP reduces state-levels of anxiety in children and thus confirmed its potential as an intervention for anxiety	Dynamical Systems Theory (DST): real-time in-game information to dynamically adjust the game environment to individual learning trajectories. Use of natrual imagery and sound to help increase relaxation. Immersive environment decreases outside distractions.
Sakamoto, M., Nakajima, T., & Alexandrova, T. (2015). Enhancing values through virtuality for intelligent artifacts that influence human attitude and behavior. Multimedia Tools and Applications; Dordrecht, 74(24), 11537–11568. doi:http://dx.doi.org.proxy.lib.sfu.ca/10.1007/s11042-014-2250-5	MikuMikuDance: AR trading card game	Supports telepresence, socialness	AR, tangibles	Projectors, motion capture, web camera	Kinect, OpenNI, Skype	gamers	N/A	input: body movement, trading cards; output: virtual character movement, augmented card projection	Real and digital combined to provide presence and keep the tactile feel of traditional trading card games. Virtual avatars are customizable for personal element.	Proof of concept	Customizable game helps to engage user. Use of cameras and projection can help users feel like they are playing with a real person, thus increasing social connection.
	Virtual Aquarium: persuasive ambient mirror that has the objective of improving users' dental hygiene by promoting correct tooth brushing habits	Promotes dental health	AR, tangibles	Accelerometer, mirror display	3-axis accelerometer sensor	humans	N/A	input: brush teeth; output: fish dance and aquarium prospers	Start to brush teeth --> scrubber cleans the aquarium; Brush --> fish start moving; Finish brushing for sufficient time --> scrubber stops and fish dance elegantly; Each day of brushing more fish are born. Don't brush and aquarium gets dirty and fish die.	Proof of concept	Six values were extracted from their case studies: 1. empathetic value - empathy for characters strengthens buy-in to experience; 2. persuasive value - offering feedback can alter user's attitudes and behaviours; 3. informative value - offering information so that the user can change; 4. economic value - tangible value that is not actually money or goods; 5. aesthetic value - using art and beauty can improve our mood and sense of well-being; 6. ideological value - increasing self-efficacy through education.
Seaborn, K. A. (2016). Mixed Reality Gaming for Older Powered Chair Users: A Human Factors Model of Well-being and Engagement (Ph.D.). Canada: University of Toronto (Canada). Retrieved from https://search-proquest-com.proxy.lib.sfu.ca/docview/1883858657/92434D3FE69845F4PQ/31	Powered to Play (PTP): capture-the-flag game that is an accessible, social mixed reality game for co-located mobile play in everyday spaces	Promotes accessable social gaming	MR	Smartphone	Android & iOS, Javascript framework (Appcelerator Titanium), Google Maps API	powered chair users	13 (8 Female)	input: physical movement; output: capture flag for points	Based on the following design recommendations: 1. multiple people with various skills and abilities can play; 2. Intergenerational gaming; 3. opportunities to meet and collaborate with new peopletoward a common goal; 4. fair playing field; 5. challenge and strategy; 6. Accessible and easy to use equipment and interface design; 7. outdoor play space; 8. opportunities to improve skills and abilities.	participants found the game tobe fun, social, and accessible, and engaged with the game in a variety of ways. Participants experienced high levelsof engagement.	observer commentary identified three complementary factors that arose as a result of the inclusive, social game design,and everyday setting: intergenerational play for multiage inclusivity and enrichment; mixed ability play that harnesses and supports the abilities of those with and without motor impairments and other disabilities; and community engagement through integration or enactment at public, open community sites.
Shaw, C. D., Gromala, D., & Seay, A. F. (2007). The meditation chamber: Enacting autonomic senses. Proc. of ENACTIVE/07.	Meditation Chamber: bio-interactive, therapeutic, virtual environment	help users lower their stress levels through meditation and muscle relaxation	VR, biofeedback	HMD, GSR, respiration belt, blood volume pulse sensor	VFX-3D (Interactive Imaging Systems), Thought Technologies ProComp+, SVE Toolkit	novice meditators	411	input: skin conductance, heart rate, respiration; output: virtual sunset, jellyfish movement, audio	visual and audio cues were used to create, guide, and maintain a user's guided relaxation and meditation	GSR levels decreased, breathing patterns were steadier and deeper, self-rated relaxation levels increased, and subjective comments were mostly positive.	Visuals and sound provided real-time feedback on performance, allowing the user to adjust their internal states. Use of abstract and natural imagery provided a means to focus on that is plesant and relaxing and decreased external distractions. Few instructions allowed for playful interaction and uninstructed learning.
Vidyarthi, J. (2012). Sonic Cradle: Evoking Mindfulness through Immersive Interaction Design (MSc Thesis). Surrey, BC, Canada: Simon Fraser University. Retrieved from https://vimeo.com/55230632	Sonic Cradle: an immersive, interactive soundscape designed for novice meditator to explore their breathing.	introduces a relaxing HCI paradigm to foster mindfulness-like meditative states	immersive soundscape, biofeedback	respiration physiological sensor, surround-sound	Max MSP, Thought Technology ProComp2	novice meditators	39 (15 Female)	Input = breathing from abdomen and chest; hold breath to add sounds, breath quickly to subtract sounds	Mindfulness practice --> Sonic Cradle Interaction<br>Calm focused attention --> focus on respiratory control through sound interaction<br>Wandering mind --> Wandering mind sounds change<br>Meta-awareness --> Autonomic breathing as attention is brought back to sounds and user wonders how their breathing controls it	Relaxation, clarity of mind, reduced thinking, emptiness	Playful, non-invasive made experience inviting. Dark room with only soundscape decreased distractions and allowed focus on breathing and present moment. Selected sounds were meditative in nature – neutral or calm yet still energetic enough to keep attention. Easy to learn mapping to explore breath. Auditory feedback on performance.
Wiethoff, A., & Butz, A. (2010). ColourVision: Controlling Light Patterns Through Postures. Proceedings of the 10th International Conference on Smart Graphics, SG'10 (pp. 281–284). Berlin, Heidelberg: Springer-Verlag. Retrieved from http://dl.acm.org/citation.cfm?id=1894345.1894384	ColourVision: people use thier posture to change the colour of a room, reflected as their current mood	people experience the psychological effects of different colors	immersive room installation	Camera, RGB lamps	C++ video analysis software, DMX converter	humans	N/A	input: posture in chair; output: room colour	The implemented body interface controls the room through posture. Red, for example, is activated through an open, active seating position. Green is the color for introverted reflectiveness as generated if a person takes a thoughtful, closed position. A person, sitting on the chair in a stretched, relaxed position plunges the room into a cool blue as the color for calmness.	Different emotional color impressions were reported from the participants: Asked about their emotional state when exposed longer to one color, for example red, the participants reported an increased level of "nervousness." An intensive exposure in a relaxed posture to the color blue was described as "peaceful."	Playful interactions with the body interface were perceived positively. Users were curious to try out different positions to see how that affected the colour. Users also reflected on their own emotions by perceiving the colour of the room. Users are shown internal states visualized and, in turn, the visuals affect the user's internal states – feedback loop
